# Assessment of potential dominant factors for brownfield landscape regeneration: A case study in Xi’an, China

**DOI:** 10.1371/journal.pone.0312668

**Published:** 2025-02-10

**Authors:** Xia Wei, Sreetheran Maruthaveeran, Mohd Fairuz Shahidan, Tao Sha

**Affiliations:** 1 Faculty of Design & Architecture, Department of Landscape Architecture, University Putra Malaysia, Serdang, Selangor Darul Ehsan, Malaysia; 2 Department of Architectural and Environmental Arts, Xi’an Academy of Fine Arts, Shaanxi, China; Vietnam Maritime University, VIET NAM

## Abstract

Rapid global urbanization has made brownfield reuse a vital issue for sustainable urban development. However, the regeneration of brownfield landscapes is a complex and lengthy process that requires a combination of factors to be considered. Their landscape regeneration must be planned and prioritized to utilize brownfield sites and achieve positive social benefits. Therefore, an urgent need must be established to establish an assessment framework and system for various types of brownfield landscape regeneration dominant factors to find different brownfield landscape regeneration dominant factors. This research developed an assessment model using the Analytic Hierarchy Process (AHP), covering five brownfield types: industrial, mining, military, transportation, and landfill in Xi’an, China. The potential assessment factors in three levels were analyzed for weighting to explore the dominant factors for the potential regeneration of brownfield landscapes in Xi’an. The results showed that, firstly, among the five first-level assessment factors, the physicality factor was the most important. Secondly, among the 16 second-level factors, the spatial and physical features of the visual landscape were the most critical. Finally, among the 40 three-level factors, spatial features were the primary factor. Therefore, the purpose of this research is to provide a specific assessment system and data analysis methods and ideas for the dominant factors of urban brownfield landscape regeneration in China and other regions based on the assessment framework with strong adaptability proposed by the AHP method, which can be flexibly adapted in the different areas and countries, to realize the sustainable development of cities in various regions.

## 1. Introduction

Urban brownfields are a global phenomenon in almost all industrialized countries [1]. These brownfields are often abandoned due to transformations in economic, spatial, or social development, for which no suitable alternative use can be found [2,3]. The concept of brownfield was gradually established in the 1990s to refer mainly to contaminated or potentially contaminated land [4]. Meanwhile, the contemporary definition of brownfields is more specific. One of its sources is the US Environmental Protection Agency, which introduced the following definition: “Abandoned, idled, or under-used industrial and commercial facilities where expansion or redevelopment is complicated by real or perceived environmental contamination” [5].

These definitions reveal that the existence of brownfields not only represents a waste of land resources but also poses serious environmental, health, and economic risks. Brownfields are often accompanied by soil and groundwater contamination, which can threaten the health of neighboring residents and increase the cost of development and use [6]. These risks add to the complexity of brownfield reuse, causing many projects to stall due to high remediation costs and regulatory requirements, affecting the surrounding area’s economic development. At the same time, the uncertainty of brownfield redevelopment poses challenges to urban planning and sustainable development, which can lead to broader environmental problems and social instability.

In China, on the other hand, with rapid industrialization and urbanization since the 1980s, enterprise transformation, out-migration, and policy closures have resulted in large amounts of idle and abandoned land in urban areas. It is estimated that more than 100,000 enterprises closed between 2001 and 2015, leaving more than 200,000 ha of high-risk brownfields [7]. The pollution problems and spatial reuse needs of these brownfields have become important issues in the sustainable development of Chinese cities. The current focus of policymakers and academics is how to effectively assess brownfields to address the environmental and health risks they pose, regenerate their spatial landscapes, and reintegrate them into the urban spatial cycle.

### 1.1 Brownfield landscape regeneration

Brownfield landscape regeneration is not only about restoring the physical aspects of the land but also involves complex ecosystem restoration, environmental pollution control, and enhancement of socio-economic benefits [8]. Therefore, when regenerating brownfield landscapes, it is necessary to combine the perspectives of different stakeholders and utilize different planning tools and methodologies to assess these factors holistically to ensure that the strategies formulated can address the unique challenges of different brownfield sites. GIS technology and community participation are essential [9]. It has been shown that perceived landscape quality influences community assessment of regeneration plans, revealing community people’s preference for post-industrial aesthetics. Therefore, brownfield regeneration schemes must consider community aspirational needs [10]. In addition, it will start with case studies and incorporate public preferences for post-industrial land transformation programs [11], establish assessment indicators for urban brownfield landscape conservation and regeneration programs, and explore comprehensive assessment methods [12] to respond to current brownfield land-scape regeneration issues. Land use typology is also a research focus for brownfield landscape regeneration, combining sustainable brownfield restoration methods with spatial and local social theories to guide brownfield conservation and regeneration [13]. Because of the diversity and complexity of urban brownfield landscape regeneration elements, urban brownfield landscape regeneration needs to be supported by multiple parties, especially the government’s participation in providing a policy framework and statistical basis for urban brownfield inventory [14].

Assessments related to brownfield landscape regeneration have focused on three types of sites: industrial and infrastructure waste sites, mining waste sites, and landfills. These sites suffer from landscape fragmentation, pollution dispersal, water disruption, and habitat degradation [15]. The core element of the brownfield “pollution” problem is “brownfield earthworks,” which emphasizes the physical and spatial dimensions [16]. Engineering and biological approaches such as toxicity and pollution management, soil improvement, revegetation, and physical remediation are available [17]. Some studies have pointed out that land near brownfields and heavily trafficked highways are more susceptible to chemical pollution [18]. Remediating contaminated soils by adopting chemical contamination by plants, such as the three autochthonous plant species *Acer pseudoplatanus* L and *Betula celtiberica* Rothm [19], is highly practicable in response to this type of contamination.

### 1.2 Assessment of brownfield landscape regeneration

Brownfield regeneration assessment studies align with the international concept of sustainable development and aim to mitigate the associated costs and risks [20]. Inter-nationally, incentives have been introduced in Germany, the UK, and France to promote sustainable brownfield regeneration [21]. Brownfield sites are complex environments with a wide range of landscape regeneration indicators, and it is crucial to summarize and generalize the indicators. The main methods of indicator selection are as follows: interviews with leaders, review of relevant literature, creation of a hierarchy of objectives, and the ability of indicators to serve the goals [22]. A framework for assessing the sustainability of brownfield development has been proposed, with the most evaluated indicators being physical, cultural, and institutional factors [23]. The UK government has attempted to elucidate whether the main differences in the treatment of brownfield sites in different regions are related to these three factors [24]. In addition, case studies of brownfield development are commonly used to generate assessment systems [25]. To reflect the integrity and health of brownfield regeneration ecosystems, a system for assessing brownfield ’ecosystem services’ (ES) has been developed, with indicators that include the provision of biologically diverse environments, the maintenance of plant and animal genetic diversity, and the quality of the soil [26]. Optimized ecosystem structures for brownfield regeneration will have sound ecological, social, and economic effects [27].

Differences between evaluators of the same speciality can affect the criteria for risk assessment and stakeholder communication. Therefore, aggregating and summarizing interprofessional and interdisciplinary perspectives is necessary to obtain objective assessment results [28]. Brownfield issues involve a combination of multi-disciplinary intersections and different research perspectives. For example, botanists have developed assessment systems to inform plant selection for regeneration in brownfield landscapes [29]. Digital technology has permeated all aspects of social development, and the development of assessment models has become famous [30]. For example, the emission of malodorous pollutants from brownfield sites can be effectively controlled using CHS-SC [31].

### 1.3 Factors for brownfield landscape regeneration

Brownfield regeneration design requires a combination of economic, environmental, and social factors [32]. The aim is to turn derelict spaces into public spaces focusing on public needs and attitudes [33,34] and the public’s ethical understanding of the changing brownfield landscape [35]. Therefore, some scholars have explored the relationship between stakeholders in sustainable brownfield regeneration by combining the Fuzzy Hierarchical Analysis (FAHP) and social network analysis (SNA) [36] and have utilized the advanced image recognition technology based on the land classification model of CNN to identify and classify urban brownfields as a means of transforming urban industrial wasteland [37], emphasizing that image and visual perception play an essential role in the regeneration and reuse of brownfields, influencing the public and decision makers’ perceptions and decisions [38]. It has also been pointed out that policies and regulations, public participation, and cooperative mechanisms are crucial factors in brownfields’ sustainable development [39]. In the assessment, the goal of sustainable urbanization and brownfield regeneration can be achieved from the combined impacts of ecological, social, and economic factors with the help of the weight-of-evidence (WOE) modeling approach of geographic information systems (GIS) [40]. This method is applied in assessing brownfield landscape regeneration and predicting the current trend of urban land becoming brownfield [41]. Brownfield landscape regeneration is related to landscape regeneration and the ecological quality of cities [42]; enhancing the multiple benefits of brownfield cleanup by applying the concept of ecosystem services highlights the importance of considering ecological services during brownfield remediation to maximize environmental and social benefits [43]. The very formation of a brownfield site determines that it has a regional historical identity and a cultural phenotype. Therefore, cultural factors are critical to brownfield regeneration [44]. The cultural characteristics of brownfield sites vary from country to country and region, so potential structures and patterns should be explored based on the contextual elements of brownfield sites in different areas [45]. Therefore, studies have addressed the importance of climate change in various regions for brownfield regeneration and the need to integrate climate considerations for sustainable brownfield regeneration [46]. Urban agriculture, as an innovative urban development model, can not only enhance the urban green space area and increase biodiversity. Some scholars focus on the integrated decision-making of urban agriculture in the gray land regeneration environment and explore how to integrate urban agriculture in the process of urban regeneration effectively [47], as well as the need to consider historical, cultural, and environmental factors in brownfield remediation for different regions and types of brownfields too retain the regional character of brownfields while promoting sustainable urban development [48].

In China, research on brownfield governance and studies is still in the early stages, and the comprehensiveness of research perspectives has yet to be developed, focusing mainly on spatial quality, environmental characteristics, and human psychological perception. Some scholars have used hierarchical analysis and GIS technology to evaluate the ecological status of waste mine areas [49,50]. There are also analytical methods such as ex-ante assessment of environmental characteristic factors of brownfields and ecosystem-related factors from a systemic perspective [51]. Some scholars, based on the case of urban brownfields, have assessed the ecological, social, and economic values of brownfields by identifying areas with potential regeneration value and summarizing methods and strategies for identifying urban rewilding opportunity spaces, preserving ecological diversity, and promoting urban sustainability, in terms of factors such as social participation, environmental vegetation, water, and climate [52,53], and analyzing the impact of brownfield space on human psychological perception from a psychological analytical approach are also widely used [54]. In addition to the previously mentioned methods, some new brownfield regeneration design methods have been proposed. For example, a novel brownfield identification method based on urban land use and business data provides more accurate and effective data support for brownfield regeneration design [55]. A quantitative and comparative study on the microclimate of landfills in China was conducted at different variable levels using remote sensing technology to explore ecological restoration in brownfield regeneration [56].

### 1.4 Comparison of AHP methodologies

AHP is a commonly used multi-criteria decision analysis method that can decompose the problem into a more understandable hierarchy of sub-problems and is suitable for dealing with complex multi-criteria decision problems. Moreover, this research on brownfield landscape regeneration involves complex and multidimensional factors and problems. Therefore, the AHP research method is chosen to help the study determine the relative importance of each factor in brownfield landscape regeneration.AHP is widely used in various fields because it effectively deals with multilevel and multifactor decision-making problems.

First of all, in applying the AHP method in industry management and decision-making, most scholars have deepened the AHP method into the FAHP and have played a key role in risk modeling and risk management. For example, describing the main characteristics of a supply chain and modeling risk assessment based on AHP and FAHP methods are practical approaches [57]. Some scholars have used these methods to develop a fuzzy model that combines the main criteria and the subjective judgment of decision-makers to select the best mining method [58]. Another study proposed an extended BBS approach that combines AHP, FAHP, and fuzzy comprehensive evaluation (FCE) to assess sustainable safety performance in the petrochemical industry [59]. In addition, other scholars have used FAHP and the entropy weighting method to combine the overall weights of design indicators and the TOPSIS method to evaluate customer value [60] comprehensively. In metro system design, the comprehensive evaluation method of metro auxiliary power system design options based on AHP and FAHP helps metro system designers and managers select the best options [61].

Secondly, in terms of building and environmental assessment applications, studies have shown that fuzzy-based assessment models play an important role in estimating the significance of structural assessment criteria for concrete buildings [62]. In addition, these models have also been used to assess and analyze the degree of seismic vulnerability related to demographic, environmental, and physical criteria [63]. Recently, the use of AHP-based methods in brownfield regeneration has gradually increased. Some scholars have used hierarchical analysis to develop landscape sustainability assessment models in South Korea and Taiwan to provide a systematic and transparent decision-making approach to promote sustainable landscape management [64] and analyze ecological restoration indicators [65,66]. To assess their relationship, matrix assessment methods combine brownfield development’s aesthetic and environmental characteristics [67]. Brownfield landscape regeneration requires government intervention and significant capital investment, so combining the environmental and socioeconomic components indicators to form an assessment system to coordinate the various interests in brownfield regeneration planning [68]. To better assess the quality of landscape design, some scholars have also used AHP to assess the spatial quality of brownfield landscapes to better utilize the social benefits of brownfield landscape regeneration [69].

In another field, the system of indicators for evaluating the health of urban aquatic ecosystems is of great importance for the assessment, management, and urban development planning of urban river networks. Studies based on AHP, FAHP, and the Interval Analytic Hierarchy Process (IAHP) aim to identify the priority indicators needed for cultural heritage management to address the complex challenges of protecting and managing cultural heritage in urban environments [70]. An important research direction is establishing an evaluation indicator system based on the relationship between people, cities, and aquatic ecosystems, covering environmental conditions, ecological construction, and social services [71]. Meanwhile, some scholars constructed a green water evaluation index system for reservoir projects based on AHP to solve the problems of unsystematic planning and designing ecological environments in reservoir basins and unbalanced ecological, economic, and social benefits [72]. In addition, related studies have developed an integrated framework to assess the importance of influencing factors and prioritize these factors to provide a sustainable management approach for the shipping industry to meet the increasing environmental challenges [73]. These studies show that applying AHP methodology in building and environmental assessment can help solve complex environmental problems and promote sustainable development.

Thirdly, applications in health assessment and information networks: For the COVID-19 epidemic, the research investigated the vaccine selection problem using a novel Vikor hybrid approach with AHP and interval type-2 fuzzy sets to cope with uncertainty [74]. The FAHP and FTOPSIS (Fuzzy Technique for Order Preference by Similarity to Ideal Solution) techniques were used to select suitable composite materials for dental restorations, providing a powerful decision-making tool for dental professionals [75]. The impact of epidemics on consumer preferences and the strategic problems FMCG companies face in selecting distribution channels have been investigated through AHP using a mixed methods approach to analyze the impact of factors on selecting distribution channels [76]. In addition, other scholars have used AHP to study the sustainability of mobile learning (CBML) in a cloud computing environment, evaluating the impact of technological, managerial, social, and environmental factors to identify critical factors for success [77].

Finally, the application in other design areas. Some researchers have studied the application of AHP and FAHP decision modeling in designing chronic disease healthcare pillboxes, and the proposed packaging design is a case study [78]. Electrotherapy designers, as well as a case study, to research the application of product fuzzy decision model selection in human factors design [79]. In intelligent backpack design, selecting suitable built-in mobile power is critical using the automatic weighted fuzzy weighted cross FAHP method to compare and determine the suitable built-in mobile power for clever backpack design [80]. Other scholars constructed an evaluation index of attractive attributes of Chinese fashion T-shirt products from the perspective of attractive consumption, an essential basis for improving product development [81].

It is easy to see from the above literature that the AHP has been widely used and discussed in various areas of multi-criteria decision-making. It stands out because of its ability to integrate qualitative and quantitative data, providing a structured framework for comparing and prioritizing factors. The advantage of AHP over other methods, such as the Delphi Method, FAHP, and Multi-Criteria Decision Analysis (MCDA), is its simplicity and ease of use. Although the Delphi method relies on iterative expert consultation to reach consensus, and the FAHP introduces fuzzy logic to deal with uncertainty in expert judgments, the direct pairwise comparisons and consistency checking of AHP make it a practical choice for many applications. Therefore, AHP was chosen for this research because of its systematic approach to dealing with the complexity of regeneration factors on brownfield sites in Xi’an. The innovations in this research application are the adaptation of the AHP framework to the unique socio-economic and cultural context of Xi’an, ensuring that the selected criteria and weights accurately reflect local conditions and priorities, and the operationalization of the methodology, which allows for the flexibility to change the content of the framework and adaptively apply it to other regions, and indeed other countries.

As summarized in the above literature, the existing research focuses on various aspects of brownfield redevelopment, and different scholars have made significant contributions to this field, especially in laying a solid foundation for brownfield landscape regeneration research. However, due to the late start of brownfield research in China. The systematic theory has yet to be formed, and most of the research has been expanded based on foreign scholars’ research, mainly focusing on exploring strategies at the theoretical level of brownfield governance. The related assessment of brownfield governance practices and specific guidance strategies are less involved, and the depth of their research needs to be improved, especially regarding the specific factors of brownfield landscape regeneration, where there still needs to be a gap. Based on the above problems, this research aims to clarify the following questions: (1) What are the main factors involved in urban brownfield landscape regeneration? Which factors are considered as potential dominant factors? (2) How do these factors compare with those in other regions? Although the focus of this research is Xi’an, China, the findings form a strategy that can be applied to cities with similar contexts, and the research forms a flexible assessment framework based on the AHP that can be adapted to other regions as well as countries with appropriate modifications, thus enhancing the applicability of this research. The above literature is summarized by the factors explicitly related to the regeneration of brownfield landscapes, as shown in Table 1.

**Table 1:** Summary of literature involving factors related to brownfield landscape regeneration.

Factors	Personal factors									Functional factors				Physical factors							Others factors	
	Age	Gender	Occupation	Education level	Socioeconomic status	Ethical /race	Family structure	length of residence	physical limitations	Safety	Sociability	Social support	Funding	Quality of space facilities	vegetation cover	pollution status (soil、water、noise、air)	Management and Maintenance	Vision&Aesthetic (architectural / landscape aesthetics)	Area accessibility	Spatial scale and enclosure (static / dynamic spatial features	Time of day/season	Climate
Loures et al.(2007) [32] PT											+	+	+	+-	+	-	+	+	+	+		
Krejčí et al.(2014) [33] CZ*	+	+	+	+		+			+-			+-	+-	+-		-	+	+	+	+ -	+	
Krejčí et al.(2016) [39] CR	+	+	+	+		+			+-			+	+	+		-	+	+ -	+	+	+	
Abdullahi et al.(2016) [40] AUS					+						+	+				-		+ -	+	+		
Mathey et al. (2018) [34] GER *	+	+		+		+	+		+-	-	+-	+	+		+	+-	+ -	+	+ -	+	+	
Feng et al.(2019) [65] CHN	+	+	+	+		+			+	+	+	+			+	-		+ -		+		
Zou (2019) [69] CHN					+				+		+	+	+	+		-	+ -	+	+	+		
Zhu, H (2019) [49] CHN											+	-			+	+	+	+	+ -	+		
Abdullahi (2021) [41] IRN											+	+				-			+	+	+	
Zhong et al.(2020) [51] CHN			+	+					+		+	+	+		+	-	+	+	+	+		
Oudes et al.(2020) [42] HL			+	+							+	+	+-		+		+	+	+	+		+
Yagci et al.(2021) [35] PT*			+	+							-	-	+-	-	+ -		+	+	+	-		
Zhu ,C et al.(2021) [54] CHN *																	+ -	+		+		
Turečková et al.(2021) [45] CR										-		-	-			-	+ -		+	+		
Szabó et al.(2022) [44] HUN *	+										+	+	+		+	-		+	+	+		
Song et al.(2022) [55] CHN *			+	+		+		+	+		+	+-	+-			+		+		+		
Wang et al.(2023) [73] CHN												+				-	+				+	+

(“+”: Positive factor; “-”: Negative factor; “+-”: Neutral factor. “*”: Key research literature).

## 2. Materials and methods

### 2.1 Site selection and sampling

This research site is located in Xi’an, Shaanxi Province, China, which is well known as the “World Famous Historical and Cultural City” and an important starting point of the Silk Road. Xi’an covers an area of more than 9,000 square kilometers and has a population of more than 7 million. According to the data, more than 3,000 acres of brownfield sites are to be developed, mainly concentrated in the western suburbs of Xi’an, and formed by the defense industry’s abandoned land.

**Fig 1:**
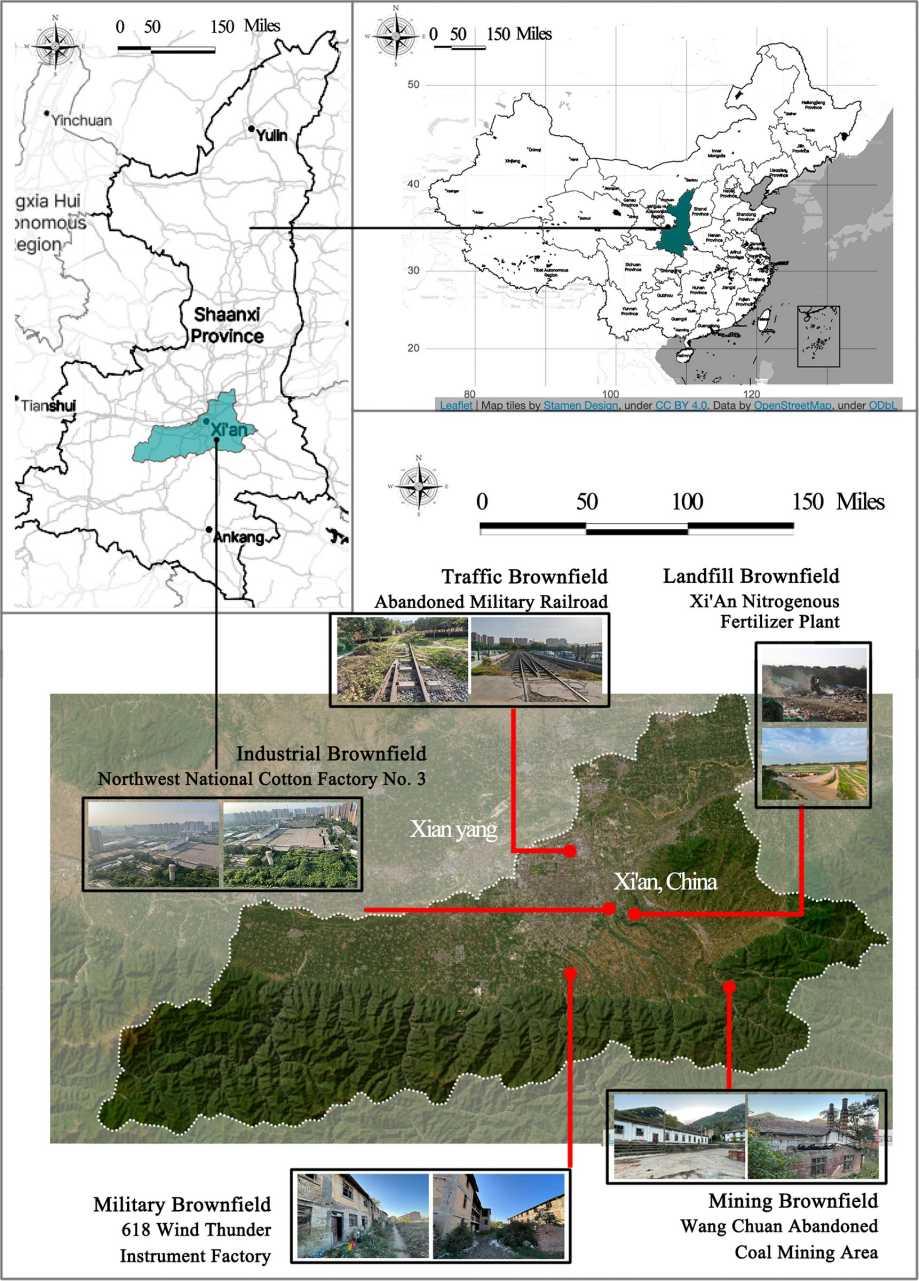
Location of Xi’an, China, main distribution areas of brownfields, and siting of five types of brownfields. The figure shows a representative selection of sites in Xi’an, China, for each of the five brownfield types. Sites from urban and suburban areas were selected to ensure a comprehensive sample. Sources of map layers are available in the S1 Appendix. A figure is similar but not identical to the original image and is for illustrative purposes only.

Five representative types of brownfield sites in Xi’an were selected for this research. These brownfields vary in character and regeneration challenges due to their historical background, geographical location, and use characteristics. They are industrial brownfields, mining brownfields, military brownfields, transportation brownfields, and landfills. It was ensured that the brownfield sites were selected to cover the city center and suburban areas to reflect the land use and development of the different regions. Second, the historical context of the brownfields was considered, including various types of industrial, military, commercial, residential, and abandoned public facilities, to ensure that the study would cover the diversity of brownfields. In addition, as this research focuses on the potential dominant factors in the regeneration of brownfield landscapes, this research mainly selected brownfield types planned to be regenerated to understand the challenges and strategies of different regeneration processes, as shown in Table 2. In terms of sampling methodology, this research used a combination of stratified and purposive sampling strategies. Through stratified sampling, this research categorized brownfield sites in Xi’an based on geographic location, historical context, and current status. This research applied purposive sampling to select brownfields with representative and research values within each category. This systematic approach finally identified the five most representative brownfields, as shown in Fig 1. These brownfield sites will provide this research with a comprehensive perspective and an in-depth understanding of the characteristics and regeneration challenges of brownfield sites in Xi’an.

**Table 2:** Characteristics and challenges of different brownfield site types.

Brownfield Type	History background	Location	Key Features	Regeneration Challenge
**Industrial Brownfield**	In the past, it was used for industrial production but was gradually abandoned with the adjustment of economic structure and industrial upgrading.	In the city or suburbs, close to transportation hubs	There may be heavy metal and chemical pollution, and environmental management of soil and groundwater is required	Environmental governance, infrastructure transformation, land use planning
**Mining Brownfield**	Used for mining in the past, abandoned when resources were exhausted or economically unfeasible	Outskirts of cities or remote areas	The terrain is broken, there may be accumulation of slag and waste, and the ecological environment is damaged	Ecological restoration, safety hazard treatment, high development costs
**Military Brownfield**	Used for military purposes in the past but abandoned as military purposes changed or bases were relocated	Urban fringe or strategic location	The facilities are well-equipped and may contain unexploded ordnance and other dangerous items	Safety inspection, facility renovation, land use conversion
**Transportation Brownfield**	In the past, it was used for transportation infrastructure but was abandoned as the transportation network was adjusted and modernized.	Near transportation hub	The facility is extensive and covers a wide area of land	Facility renewal, land consolidation, and community integration
**Landfill**	In the past, it was used as a landfill but was abandoned or transformed as cities developed and environmental requirements increased.	Urban fringe or suburbs	The land is covered with garbage, which may cause harmful gas emissions such as methane	Pollution control, land restoration, long-term monitoring

### 2.2 Data collection

According to the characteristics and steps of the AHP research method, the data collection of this research mainly comes from the assessment weight values of relevant experts for each factor. To allow all invited experts to understand the current status of brownfield sites in detail and make an objective evaluation, and to ensure the accuracy and reliability of the assessment data for the five types of brownfield sites, this research used a variety of means of data collection, mainly on-site photographs and text description, questionnaire design, and semi-structured interviews with expert samples.

#### 2.2.1 Photography

The purpose of on-site photography is to provide experts with comprehensive and accurate information to ensure they can make more objective and scientific decisions during the assessment process. In the study of the five brownfield types selected in Xi’an, this research conducted on-site photography and in-depth research to ensure that the experts had a clear understanding of the current status of each site when evaluating the weights. The authors have taken many photographs of each brownfield type and have described and documented their geographic location, topography, vegetation cover, water conditions, contamination, and ecological damage to provide informative and essential information for qualitative research. In addition, this research analyzed and studied each site’s coverage, historical background, socio-economic conditions, and environmental problems, as shown in Table 3. These site research data provide an essential reference basis for the subsequent brownfield management and ecological remediation work.

**Table 3:** Specific information on the brownfield study sample.

Site Name	Brownfield Types	Location	Area	History background	Site status &Management
**①Northwest National Cotton Factory** **No. 3**	Industrial Brownfield	Baqiao District of Xi’An (Urban area)	≈136784㎡	The site is mainly abandoned, and the north side has been transformed into an industrial arts district. Pollution.	·Site status: Flat terrain, good plant cover, less water pollution. ·Management: Managed, to be developed.
**②** **Wang** **Chuan Abandoned Coal Mining Area**	Mining Brownfield	Lantian County in Xi’An (Suburbia)	≈2587312㎡	The site has been abandoned and unmanaged until now.	·Rugged terrain, good plant cover, high water pollution. · Management: Managed, to be developed.
**③** **618 Wind Thunder Instrument Factory**	Military Brownfield	Chang’An District of Xi’An (Suburbia)	≈36397786 ㎡	Bankruptcy in 2006, abandoned until now, a small portion converted into an art district	· Flat terrain, poor plant cover, less water pollution. ·Management: Unmanaged and abandoned.
**④** **Abandoned Military Railroad**	Traffic Brownfield	Weiyang Daxing New District (Urban area)	≈74800㎡	Site railroad tracks have been removed and are awaiting rezoning.	· Flat terrain, poor vegetation cover, and high water pollution. ·Management: Unmanaged and abandoned.
**⑤** **Xi’an Jiangcun Landfill**	Landfill	Baqiao District of Xi’an (Suburbia)	≈349200㎡	It was built in 1994. Recently, the park was closed.	·High terrain, poor vegetation cover, and water pollution. ·Management: Strict management and initial treatment of part of the site.

#### 2.2.2 Questionnaire

A questionnaire survey is a standard quantitative research method widely used in various fields such as social sciences, market research, and academic research because of its ability to collect a large amount of data quickly and accurately. Based on the field research, this research designed an AHP questionnaire containing 27 questions covering the comprehensive assessment factors from A to D. During the questionnaire design process, relevant literature and theories were reviewed, and several revisions and optimizations were made based on the feedback from experts invited in advance to ensure the accuracy and feasibility of the questionnaire design. The questionnaire was distributed to relevant experts both on-site and by mail. During the distribution process, extensive communication and explanations were made with the experts to ensure that they understood the content and purpose of the questionnaire.

#### 2.2.3 Expert sample interviews

Semi-structured interviews offer great flexibility and depth, allowing for a better understanding of experts’ views and perspectives. According to international research standards, using the AHP method, it is usually recommended that a sample size of 15-20 experts be collected. Semi-structured interviews were used in this research, and 20 representative experts were selected to participate. These experts’ professional backgrounds cover Landscape design, Architectural design, Urban planning, and Historic building preservation, among others. In choosing the experts, first of all, the experts need to have an advanced degree (such as a master’s or a Ph.D.) and have more than five years of practical work experience in these fields. In addition, they have experience in brownfield regeneration, urban planning, or related projects and hold academic qualifications or industry certifications (e.g., registered architect, landscape architect). These criteria ensure diversity and specialization of experts, thus reducing potential bias.

This research used semi-structured face-to-face interviews and online and offline survey data collection methods to collect 20 valid questionnaires (12 online and 8 offline). The two data collection methods, interviews and questionnaires, were chosen for this study because the questionnaires were mainly used to collect experts’ quantitative evaluation of the importance of each factor. In contrast, the interviews explored experts’ in-depth understanding of the current status of brownfield sites and related regeneration factors. Such an approach reflects the diversity of data collection methods and the rigor of the assessment but also provides more comprehensive and objective information for this study, which is an essential reference for the subsequent decision-making analysis of potential dominant factors in brownfield landscape regeneration. The detailed professional breakdown of the experts and the necessary information on their academic background are shown in Table 4.

**Table 4:** Background information of the experts.

Items	Related details	Percentage
**Gender**	Man	60%
	Female	40%
**Age**	35-45	20%
	45-55	35%
	55-65	40%
	Over 65	5%
**Education**	Junior college	5%
	Bachelor’s Degree	45%
	Master’s Degree	30%
	Doctoral Degree	20%
**Major**	Landscape design	25%
	Architectural design	15%
	Landscape architecture	25%
	Urban planning	10%
	Historic building preservation	5%
	Civil engineering	5%
	Community and housing planning	5%
	Engineering management	5%
	Structural engineering	5%
**Workplace**	Design institute	30%
	Architectural design firms	10%
	Government agency	30%
	Academic institution	20%
	Enterprise management	10%

### 2.3 AHP implementation

The AHP is a system for evaluating complex problems that can be broken down into multiple levels and elements for decision-making and assessment. Its features include systematization, transparency, and repetition, eliminating subjective ambiguity and public scepticism; thus, it is widely used in sustainable development and other fields. The implementation process includes establishing a hierarchical model of assessment indicators for the target problem, defining assessment indicator scales and meanings, inviting experts to conduct an assessment, calculating weights and obtaining priorities, synthesizing and ranking the weights, and finally obtaining preferred options. Meanwhile, the results of AHP analysis can be visualized using SPSS and EXCEL software. This research combines the characteristics of five types of brownfield sites in Xi’an, establishes a spatial assessment system for brownfield landscape regeneration in Xi’an, uses the hierarchy analysis method to compare the components of spatial quality according to the hierarchy, and makes an order of importance, and ultimately establishes the weighting and ordering of the elements of each hierarchy, to obtain the priority options for brownfield landscape regeneration in Xi’an. The AHP The detailed data analysis steps are as follows:

#### 2.3.1 Assessment indicator hierarchy model

**Fig 2:**
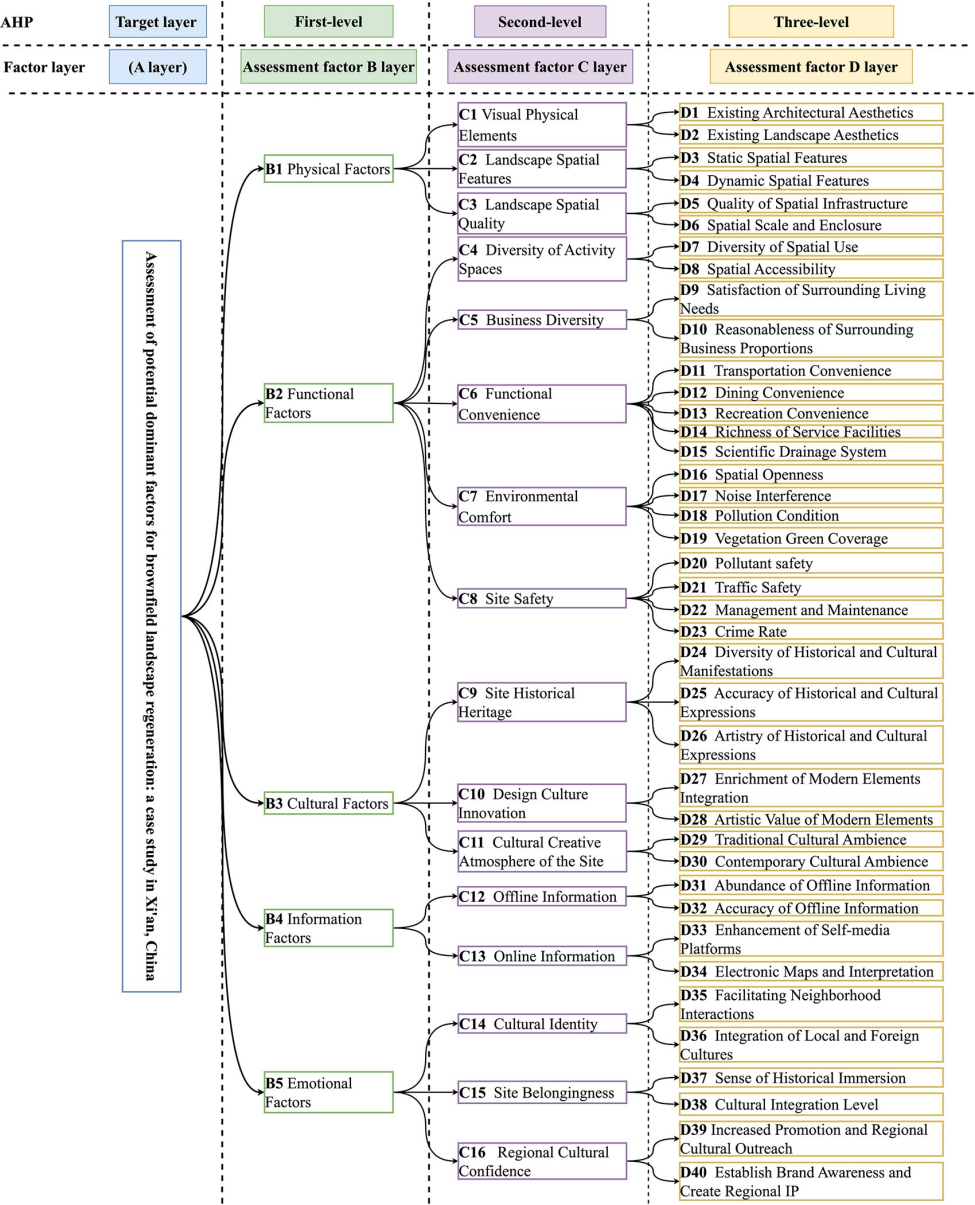
Assessment indicator AHP model for brownfield landscape regeneration in Xi’an.

Based on the current research data, this research searched for relevant literature to establish a comprehensive evaluation of brownfield sites in Xi’an and invited five landscape and planning experts to conduct semi-structured face-to-face interviews to establish a four-tiered evaluation hierarchy: i.e., the objective layer (A), the first-tier assessment factor layer (B1-B5), the second-tier assessment factor layer (C1-C16), and the third-tier assessment factor layer (D1-D40), and the assessment factors pointing to a gradual clarification of the assessment factors from A to D. The assessment factors point to a gradual clarification of the assessment factors. A to D layer assessment factors point to gradual clearing. The assessment indicator hierarchy model for brownfield landscape regeneration in Xi’an is given in Fig 2.

#### 2.3.2 Definition of assessment indicator scales and meanings

Based on Fig 2, the AHP model was utilized with a nine-point scale to establish the basis for pairwise comparisons between the assessment factor layers and assess the relative importance of the factors. The scale of the nine-level scaling method and its definition are shown in Table 5, where B_i_ and B_j_ are the same levels of assessment factors; B_ij_ is the importance comparison between B_i_ and B_j_, and B_ji_ is the importance comparison between B_j_ and B_i_; i, j=1, 2, …, n, “n” is the number of assessment factors existing in the current level. For example, B_i_ is slightly more important than B_j_, and according to Table 5, B_ij_ = 3 and B_ji_ = 1/3.

**Table 5:** Specific scales and meanings of the nine-layer assessment scale.

Assessment scale (B_ij_)	Definition
**1**	B_i_ and B_j_ are equally important
**3**	B_i_ is slightly more important than B_j_
**5**	B_i_ is a little more important than B_j_
**7**	B_i_ is much more important than B_j_
**9**	B_i_ than B_j_ is absolutely important
**2, 4, 6, 8**	In the middle of 1,3,5,7,9

B_i_ compared to B_j_, B_ij_ = 1/B_ji_; assessment factors compared to themselves, B_ij_ = B_ji_ = 1.

#### 2.3.3 Pairwise comparison and assessment of factors at each level

Assuming “n” elements, each element is compared with other elements to form an “n × n” matrix. Experts, through the AHP evaluation model with the corresponding evaluation scale, fill in the corresponding values according to the importance of the factors to get each factor’s weight. For example, a pairwise comparison matrix is constructed based on the three-factor layers i, J, and K, as shown in Table 6.

**Table 6:** Pairwise comparative evaluation framework.

	I	J	K
**I**	1	a	b
**J**	1/a	1	c
**K**	1/b	1/c	1

In this matrix, “a” denotes the relative importance of I relative to J; “b” denotes the relative importance of I relative to K. “c” represents the relative importance of J relative to K.

#### 2.3.4 Testing for consistency and calculating factor weights

Based on the nine-level assessment scale, 20 experts rated the factors. They calculated the average value, resulting in an assigned value for each assessment factor, with the average value retained in whole numbers. However, due to differences like work, professional background, and cognition among the experts, as well as differences in the experts’ understanding of the different assessment factors, there may be logical errors in recognizing the degree of importance among the assessment factors. For example, there may be cases where “a” is more critical than “b”, “b” is more essential than “c”, and “c” is more important than “a”. Therefore, consistency testing (CR) is required to ensure the accuracy and reliability of assessment results [82]. When CR<0.1. It indicates that the pairwise comparison matrix satisfies the consistency test. Conversely, if CR>0.1, it suggests that the pairwise comparison matrix needs adjustment.

Calculating factor weights and priority ranking is the final step in the AHP. Firstly, the pairwise comparison matrices are normalized. For example, assuming the element’s weight in a row “i” and column “k”, the calculation formula is shown in Eq (1). Following this approach, the pairwise comparison matrices are calculated column-wise to obtain normalized weights. Secondly, several averaging methods are employed to calculate the arithmetic mean, resulting in row weights “W_i_”. Finally, by synthesizing the row weights “W_i_” and column weights “W_j_”, the total weight “W_T_” is obtained, which determines the ranking of element weights, as shown in Eq (2). The entire process is analyzed using SPUSS software and visualized for better comprehension.


$$\left\{ \begin{array}{c} \omega_{ik}=\frac{a_{ik}}{\sum_{j=1}^{n}\;a_{ij}}\\ \omega_{i}=\frac{1}{n}\sum_{k=1}^{n}\;\omega_{ik} \end{array}\right.$$ (1)

In this equation, “n” represents the current evaluation factors: i, j, k = 1, 2, …, n.


$${\omega_{T}=\omega }_{i}\omega_{j}$$ (2)

In this equation, “W_i_” represents the row weights; “W_j_” represents the column weights: i, j, k = 1, 2, …, n.

## 3. Results

### 3.1 Weight of LayerB relative to the layer A

**Fig 3:**
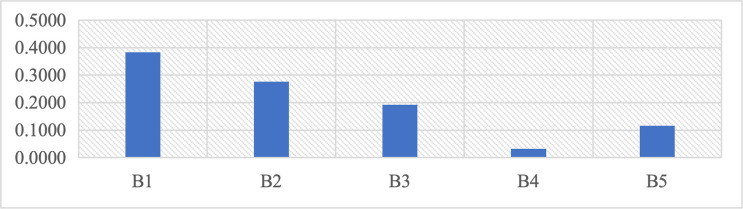
Weights of the first-level assessment factors in Layer B.

According to the data analysis in Table 7 and Fig 3, the physical factor B1 (0.3829) occupies the most critical position in the first level assessment factor B layer of landscape regeneration of brownfield sites in Xi’an. Functional factor B2 (0.2759) is slightly more critical than cultural factor B3 (0.1928). It is followed by emotional factors B5 (0.1162) and informational factors B4 (0.0322). The results show that in the strategy of regenerating these five types of brownfield landscapes in Xi’an, the physical characteristics of the brownfield sites should be emphasized to highlight the characteristics of the sites. Then, the essential functions should be implemented according to public demand. At the same time, the regional cultural characteristics of the region where the brownfield sites are located should be inherited to continue the historical and cultural genes of the sites.

**Table 7:** Pairwise comparison matrix for correspondence between Layer B and Layer A.

A	B1	B2	B3	B4	B5	Weights (*W*_*i*_)
**B1**	1	2	2	9	3	0.3829
**B2**	1/2	1	2	7	3	0.2759
**B3**	1/2	1/2	1	7	2	0.1928
**B4**	1/9	1/7	1/7	1	1/5	0.0322
**B5**	1/3	1/3	1/2	5	1	0.1162
**vector of priorities** (*v*)	2.551	1.838	1.285	0.214	0.774	
*λ*_*max*_ *= 5*.*120*, *CI = 0*.*030*, *RI = 1*.*120*, *CR = 0*.*027<0*.*1*, Satisfy the consistency test.						

### 3.2 Weight of the Layer C relative to the Layer B

According to the second level assessment factor C tier shown in Table 8, the factors C1-C7 are subdivisions of the first level assessment factor B tier. Among the B1 physical factors, landscape spatial features C2 (0.6491) have a critical status and occupy a dominant position. Among the B2 functional factors, environmental comfort C7 and site safety C8 have equal importance, accounting for (0.3514) respectively, followed by functional convenience C6 (0.1818). In B3 cultural factors, site historical heritage C9 (0.4934) and design cultural innovation C10 (0.3108) are more critical than the cultural creative atmosphere of the site C11 (0.1958), especially to highlight the site historical heritage. In the B4 information factors, offline information C12 (0.5000) is as essential as C13 (0.5000) online information. Finally, in B5 emotional factors, compared to the regional cultural confidence C16 (0.1692), the cultural identity C14 (0.4434) and C15 site belongingness (0.3874) are more critical, especially the sense of cultural identity.

**Table 8:** Pairwise comparison matrix for correspondence between Layer C and Layer B.

B1	C1	C2	C3			Weights (*W*_*i*_)
**C1**	1	1/3	5			0.2790
**C2**	3	1	7			0.6491
**C3**	1/5	1/7	1			0.0719
**vector of priorities (*v***)	1.186	2.759	0.306			
*λ*_*max*_ *= 3*.*065*, *CI = 0*.*032*, *RI = 0*.*520*, *CR = 0*.*062<0*.*1*, *Satisfy the consistency test*.						
**B2**	**C4**	**C5**	**C6**	**C7**	**C8**	**Weights (** *W*_*i*_ **)**
**C4**	1	3	1/5	1/5	1/5	0.0703
**C5**	1/3	1	1/5	1/5	1/5	0.0453
**C6**	5	5	1	1/3	1/3	0.1818
**C7**	5	5	3	1	1	0.3514
**C8**	5	5	3	1	1	0.3514
**vector of priorities (*v*)**	0.474	0.306	1.227	2.371	2.371	
*λ*_*max*_ *= 5*.*347*, *CI = 0*.*087*, *RI = 1*.*120*, *CR = 0*.*077<0*.*1*, Satisfy the consistency test.						
**B3**	**C9**	**C10**	**C11**			**Weights (** *W*_*i*_ **)**
**C9**	1	2	2			0.4934
**C10**	1/2	1	2			0.3108
**C11**	1/2	1/2	1			0.1958
**vector of priorities (*v*)**	1.587	1.000	0.630			
*λ*_*max*_ *=3* .*054*, *CI = 0*.*027*, *RI = 0*.*520*, *CR = 0*.*052<0*.*1*, Satisfy the consistency test.						
**B4**	**C12**	**C13**				**Weights (** *W*_*i*_ **)**
**C12**	1	1				0.5000
**C13**	1	1				0.5000
**vector of priorities (*v*)**	1.000	1.000				
*λ*_*max*_ *= 2*.*000*, *CI = 0*.*000*, *RI = 0*.*00*, *CR = null*, *Satisfy the consistency test*.						
**B5**	**C14**	**C15**	**C16**			**Weights (** *W*_*i*_ **)**
**C14**	1	1	3			0.4434
**C15**	1	1	2			0.3874
**C16**	1/3	1/2	1			0.1692
**vector of priorities (*v*)**	1.142	1.260	0.550			
*λ*_*max*_ *= 3*.*018*, *CI = 0*.*009*, *RI = 0*.*520*, *CR = 0*.*018<0*.*1*, Satisfy the consistency test.						

**Fig 4:**
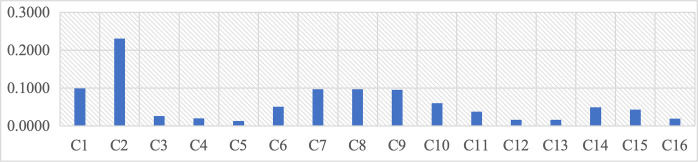
Comprehensive weight of Layer C in second-level assessment factors.

According to the results of Table 9 and Fig 4, the total weights and ranking data of the C-level assessment factors show that landscape spatial features C2 (0.2309) and visual physical elements C1(0.0993) belonging to the B1 physical factors occupy the most important positions. Next are the environmental comfort C7 (0.0970) and site safety C8 (0.0970) of the B2 functional factors, and the site historical heritage C9 (0.0951) and design culture innovation C10 (0.0599) of the B3 cultural factors. Due to the more excellent categorization of the B2 functional factors, its weight score in the C-level indicators has been reduced, but its importance is still maintained. Among the 16 C-level assessment factors, relatively weak importance is reflected in C5 (0.0125) business diversity, C12 (0.0162) offline information, C13 (0.0162) online information, as well as C16 (0.0187) regional culture confidence, and C4 (0.0194) diversity of activity space. These data provide specific assessment indexes and references for the landscape regeneration of class 5 brownfield sites in Xi’an.

**Table 9:** Overall weight and ranking of Layer C in AHP.

A	B1 ——— 0.3829	B2 ——— 0.2759	B3 ——— 0.1928	B4 ——— 0.0322	B5 ——— 0.1162	Total weights (*W*_*T*_)	Rank
**C1**	0.2790					0.0993	2
**C2**	0.6491					0.2309	1
**C3**	0.0719					0.0256	10
**C4**		0.0703				0.0194	11
**C5**		0.0453				0.0125	14
**C6**		0.1818				0.0502	6
**C7**		0.3514				0.0970	3
**C8**		0.3514				0.0970	3
**C9**			0.4934			0.0951	4
**C10**			0.3108			0.0599	5
**C11**			0.1958			0.0378	9
**C12**				0.5000		0.0162	13
**C13**				0.5000		0.0162	13
**C14**					0.4434	0.0490	7
**C15**					0.3874	0.0428	8
**C16**					0.1692	0.0187	12

### 3.3 Weight of the Layer D relative to the Layer C

Assessment factor D is the last level of the assessment system of five types of brownfield landscape regeneration dominant factors in Xi’an, and the assessment factors are specific to all aspects of the design elements, with a total of 40 indicators. As shown in Table 10, the assessment options are relatively simple compared to the C level, mostly with two choices, and the assessment is mainly based on the grades of “equally important” and “slightly important.” However, the four elements of C6 functional convenience, C7 environmental comfort, C8 site safety, and C9 site historical heritage have more assessment options, so the weighting indicators are more diverse. In C6 functional convenience, D13 (0.2979) recreation convenience, and D14 (0.2979) richness of service facilities are more critical. In C7 Environmental Comfort, D19 (0.4220) vegetation green coverage and D18 (0.3991) pollution condition are the primary assessment indicators. In C8 site safety, D20 (0.4921) pollutant safety and D21 (0.2320) traffic safety are more critical. Finally, the most crucial part of the C9 site’s historical heritage is the D24 (0.4579) diversity of historical and cultural manifestations and the D25 (0.4161) accuracy of historical and cultural expressions.

**Table 10:** Pairwise comparison matrix for correspondence between Layer D and Layer C.

C1	D1	D2					Weights (*W*_*i*_)
**D1**	1	3					0.7500
**D2**	1/3	1					0.2500
**vector of priorities (***v*)	1.732	0.577					
*λ*_*max*_ *= 2*.*000*, *CI = 0*.*000*, *RI = 0*.*00*, *CR = null*, Satisfy the consistency test.							
**C2**	**D3**	**D4**					**Weights (** *W*_*i*_ **)**
**D3**	1	5					0.8333
**D4**	1/5	1					0.1667
**vector of priorities (***v*)	2.236	0.447					
*λ*_*max*_ *= 2*.*000*, *CI = 0*.*000*, *RI = 0*.*00*, *CR = null*, Satisfy the consistency test.							
**C3**	**D5**	**D6**					**Weights (** *W*_*i*_ **)**
**D5**	1	3					0.7500
**D6**	1/3	1					0.2500
**vector of priorities (*v***)	1.732	0.577					
*λ*_*max*_ *= 2*.*000*, *CI = 0*.*000*, *RI = 0*.*00*, *CR = nul*l, Satisfy the consistency test.							
**C4**	**D7**	**D8**					**Weights (** *W*_*i*_ **)**
**D7**	1	5					0.8333
**D8**	1/5	1					0.1667
**vector of priorities (***v*)	2.236	0.447					
*λ*_*max*_ *= 2*.*000*, *CI = 0*.*000*, *RI = 0*.*00*, *CR = null*, Satisfy the consistency test.							
**C5**	**D9**	**D10**					**Weights (** *W*_*i*_ **)**
**D9**	1	2					0.6667
**D10**	1/2	1					0.3333
**vector of priorities (***v*)	1.414	0.707					
*λ*_*max*_ *= 2*.*000*, *CI = 0*.*000*, *RI = 0*.*00*, *CR = null*, Satisfy the consistency test.							
**C6**	**D11**	**D12**	**D13**	**D14**	**D15**		**Weights (** *W*_*i*_ **)**
**D11**	1	2	1/2	1/2	1		0.1578
**D12**	1/2	1	1/3	1/3	1/2		0.0885
**D13**	2	3	1	1	2		0.2979
**D14**	2	3	1	1	2		0.2979
**D15**	1	2	1/2	1/2	1		0.1578
**vector of priorities (*v***)	0.871	0.488	1.644	1.644	0.871		
*λ*_*max*_ *=5*.*013*, *CI = 0*.*003*, *RI = 1*.*120*, *CR = 0*.*003<0*.*1*, Satisfy the consistency test.							
**C7**	**D16**	**D17**	**D18**	**D19**			**Weights (** *W*_*i*_ **)**
**D16**	1	1/3	1/4	1/5			0.0678
**D17**	3	1	1/5	1/5			0.1111
**D18**	4	5	1	1			0.3991
**D19**	5	5	1	1			0.4220
**vector of priorities (***v*)	0.359	0.589	2.115	2.236			
*λ*_*max*_ *= 4*.*191*, *CI = 0*.*064*, *RI = 0*.*890*, *CR = 0*.*072<0*.1, Satisfy the consistency test.							
**C8**	**D20**	**D21**	**D22**	**D23**			**Weights (** *W*_*i*_ **)**
**D20**	1	3	3	3			0.4921
**D21**	1/3	1	2	2			0.2320
**D22**	1/3	1/2	1	1			0.1379
**D23**	1/3	1/2	1	1			0.1379
**vector of priorities (*v***)	2.280	1.075	0.639	0.639			
*λ*_*max*_ *= 4*.*060*, *CI = 0*.*020*, *RI = 0*.*890*, *CR = 0*.*023<0*.*1*, Satisfy the consistency test.							
**C9**	**D24**	**D25**	**D26**				**Weights (** *w*_*i*_ **)**
**D24**	1	1	4				0.4579
**D25**	1	1	3				0.4161
**D26**	1/4	1/3	1				0.1260
**vector of priorities (*v***)	1.587	1.442	0.437				
*λ*_*max*_ *= 3*.*009*, *CI = 0*.*005*, *RI = 0*.*520*, *CR = 0*.*009<0*.*1*, Satisfy the consistency test.							
**C10**	**D27**	**D28**					**Weights (** *W*_*i*_ **)**
**D27**	1	1					0.5000
**D28**	1	1					0.5000
**vector of priorities (***v*)	1.000	1.000					
*λ*_*max*_*=2*.*000*, *CI = 0*.*000*, *RI = 0*.*00*, *CR = null*, Satisfy the consistency test.							
**C11**	**D29**	**D30**					**Weight (** *W*_*i*_ **)**
**D29**	1	1					0.5000
**D30**	1	1					0.5000
**vector of priorities (***v*)	1.000	1.000					
*λ*_*max*_ *= 2*.*000*, *CI = 0*.*000*, *RI = 0*.*00*, *CR = null*, Satisfy the consistency test.							
**C12**	**D31**	**D32**					**Weights (** *W*_*i*_ **)**
**D31**	1	1					0.5000
**D32**	1	1					0.5000
**vector of priorities (***v*)	1.000	1.000					
*λ*_*max*_*=2*.*000*, *CI = 0*.*000*, *RI = 0*.*00*, *CR = null*, Satisfy the consistency test.							
**C13**	**D33**	**D34**					**Weights (** *W*_*i*_ **)**
**D33**	1	2					0.6667
**D34**	1/2	1					0.3333
**vector of priorities (***v*)	1.414	0.707					
*λ*_*max*_ *= 2*.*000*, *CI = 0*.*000*, *RI = 0*.*00*, *CR = null*, Satisfy the consistency test.							
C14	**D35**	**D36**					**Weights (** *W*_*i*_ **)**
D35	1	3					0.7500
D36	1/3	1					0.2500
vector of priorities **(***v*)	1.732	0.577					
*λ*_*max*_ *= 2*.*000*, *CI = 0*.*000*, *RI = 0*.*00*, *CR = null*, Satisfy the consistency test.							
**C15**	**D37**	**D38**					**Weights (** *W*_*i*_ **)**
**D37**	1	1					0.5000
**D38**	1	1					0.5000
**vector of priorities (***v*)	1.000	1.000					
*λ*_*max*_ *= 2*.*000*, *CI = 0*.*000*, *RI = 0*.*00*, *CR = null*, Satisfy the consistency test.							
**C16**	**D39**	**D40**					**Weights (** *W*_*i*_ **)**
**D39**	1	1					0.5000
**D40**	1	1					0.5000
**vector of priorities (***v*)	1.000	1.000					
*λ*_*max*_ *= 2*.*000*, *CI = 0*.*000*, *RI = 0*.*00*, *CR = null*, Satisfy the consistency test.							

**Fig 5:**
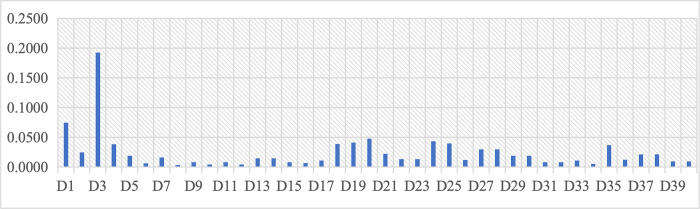
Comprehensive weight of Layer D in Third-level assessment factors.

According to the data on the combined weights of level D shown in Table 11 and Fig 5, there are three overall classes. First, the high-level comprehensive weight data mainly includes D3 (0.1924) static spatial features and D1 (0.0745) existing architectural aesthetics. Second, the comprehensive weight data of medium grade mainly includes D20 (0.0477) Pollutant safety, D24 (0.0435) Diversity of historical and cultural manifestations, D19 (0.0409) vegetation green coverage, D25 (0.0396) accuracy of historical and cultural expression, D18 (0.0387) pollution condition, D4 (0.0385) dynamic spatial features, and D35 (0.0368) facilitating neighborhood interactions. Finally, the comprehensive weighted data of the low grade mainly include D8 (0.0032) spatial accessibility, D10 (0.0042) the reasonableness of surrounding business proportions, D12 (0.0044) dining convenience, and D34 (0.0054) electronic map and interpretation.

**Table 11:** Overall weight and ranking of Layer D in AHP.

D	C1 —— 0.0993	C2 —— 0.2309	C3 —— 0.0256	C4 —— 0.0194	C5 —— 0.0125	C6 —— 0.0502	C7 —— 0.0970	C8 —— 0.0970	Total weights (WT)	Rank
**D1**	0.7500								0.0745	2
**D2**	0.2500								0.0248	11
**D3**		0.8333							0.1924	1
**D4**		0.1667							0.0385	8
**D5**			0.7500						0.0192	14
**D6**			0.2500						0.0064	27
**D7**				0.8333					0.0162	16
**D8**				0.1667					0.0032	31
**D9**					0.6667				0.0083	23
**D10**					0.3333				0.0042	30
**D11**						0.0824			0.0079	25
**D12**						0.1594			0.0044	29
**D13**						0.1594			0.0150	17
**D14**						0.5492			0.0150	17
**D15**						0.0497			0.0079	25
**D16**							0.0678		0.0066	26
**D17**							0.1111		0.0108	21
**D18**							0.3991		0.0387	7
**D19**							0.4220		0.0409	5
**D20**								0.4921	0.0477	3
**D21**								0.2320	0.0225	12
**D22**								0.1379	0.0134	18
**D23**								0.1379	0.0134	18
D	C9 —— 0.0993	C10 —— 0.2309	C11 —— 0.0256	C12 —— 0.0194	C13 —— 0.0125	C14 —— 0.0502	C15 —— 0.0970	C16 —— 0.0970	Total weights (WT)	Rank
**D24**	0.4579								0.0435	4
**D25**	0.4161								0.0396	6
**D26**	0.1260								0.0120	20
**D27**		5000							0.0300	10
**D28**		0.5000							0.0300	10
**D29**			0.5000						0.0189	15
**D30**			0.5000						0.0189	15
**D31**				0.5000					0.0081	24
**D32**				0.5000					0.0081	24
**D33**					0.6667				0.0108	21
**D34**					0.3333				0.0054	28
**D35**						0.7500			0.0368	9
**D36**						0.2500			0.0123	19
**D37**							0.5000		0.0214	13
**D38**							0.5000		0.0214	13
**D39**								0.5000	0.0094	22
**D40**								0.5000	0.0094	22

Among the high-grade assessment indicators, the main focus is on the B1 physical factors layer; among the medium-grade assessment indicators, the main focus is on the B2 functional factors layer and the B3 cultural factors layer, which cover medium and low grades because of more subdivided levels, but still perform as medium grades in general. The B4 informational and B5 emotional factors layers are concentrated in the low grade, especially in the B4 assessment factors layer.

### 3.4 Comparison and analysis of importance ratings of experts in different specialties

**Fig 6:**
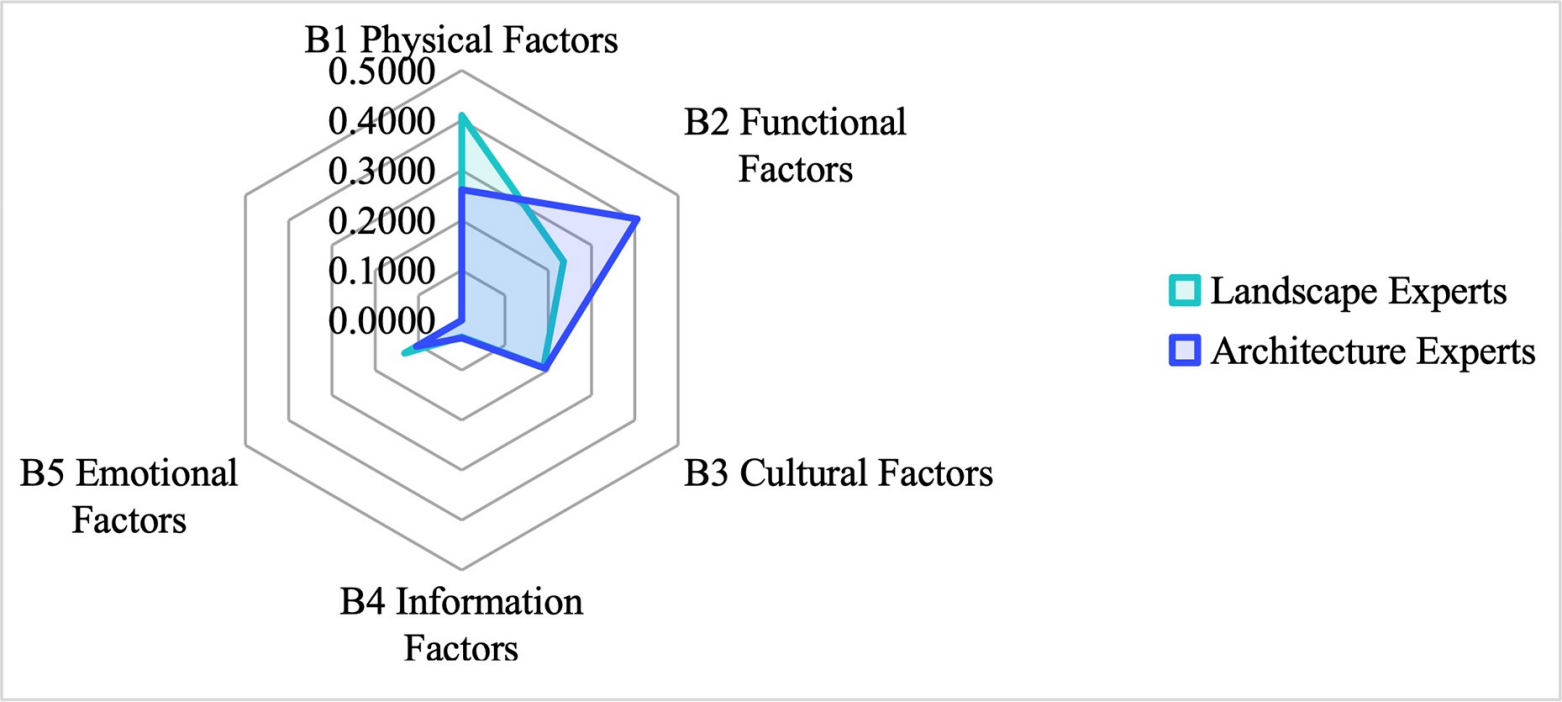
Differences in importance assessment among different experts. The figure shows the difference between the weighting and ranking of the landscape and architecture experts in selecting dominant factors.

Table 12 and Fig 6 compare the ranking of the B-level assessment factors between the 20 experts with landscape and architectural backgrounds. The results show that landscape experts ranked “B1 Physical Factors” as the dominant factor for landscape regeneration of five brownfield sites in Xi’an. In contrast, architectural experts ranked “B2 Functional Factors” as the dominant factor. There was no difference in the ranking of the other factors.

**Table 12:** Comparison of ranking of Factors B by landscape experts and architecture experts.

Rank	Total (N=20)		Landscape experts (N=10)		Architecture Experts (N=10)	
	Assessment of Factor B	Weights	Assessment of Factor B	Weights	Assessment of Factor B	Weights
**1**	B1 physical factors	0.3829	B1 physical factors	0.4100	B2 functional factors	0.4055
**2**	B2 functional factors	0.2759	B2 function factors	0.2355	B1 physical factors	0.2613
**3**	B3 cultural factors	0.1928	B3 cultural factors	0.1890	B3 cultural factors	0.1928
**4**	B5 emotional factors	0.1162	B5 emotional factors	0.1321	B5 emotional factors	0.1048
**5**	B4 information factors	0.0322	B4 information factors	0.0334	B4 information factors	0.0355
	*CR=0*.*027<0*.*1*		*CR=0*.*038<0*.*1*		*CR=0*.*040<0*.*1*	

The reason behind these differences is that landscape experts are concerned with aesthetics, ecological balance, and environmental sustainability and hope to enhance the visual appeal and ecological health of brownfield sites through physical modifications such as green space, water bodies, and environmental remediation so that they can coexist in harmony with the nature of the city. Architectural experts, on the other hand, focus more on functional factors, paying attention to architectural design, infrastructure development, and adaptive reuse of existing structures to ensure that the transformed brownfield site meets the city’s real needs, such as housing, commercial, and public facility provision, thus enhancing the project’s economic viability and long-term sustainability. In addition, prioritizing functional considerations ensures that brownfield regeneration projects are effectively connected to the city’s overall transportation network and public services, enhancing the region’s economic vitality and social cohesion. Despite these differences, the consensus between the two experts on cultural, informational, and emotional factors suggests that successful brownfield regeneration requires a combination of aesthetic, functional, cultural, and socio-economic considerations to achieve sustainable urban regeneration. The diversity of perspectives within the field highlights the complexity and interdisciplinary nature of brownfield regeneration efforts. It emphasizes integrating various perspectives to achieve holistic and sustainable urban development.

## 4. Discussion and case studies

### 4.1 Selection of factors related to brownfield landscape regeneration

Based on the characteristics of brownfield sites in Xi’an, China, this research combines a large number of literature reviews and expert interviews to identify five key factors such as physical, functional, cultural, informational, and emotional and constructs a framework for brownfield landscape regeneration assessment using the AHP method. This framework responds effectively to contemporary brownfield issues’ diverse needs and challenges and ensures that regeneration efforts are culturally sensitive and community-oriented. However, this does not mean that other unrelated factors are not necessary. Due to data limitations, potential political, economic, technological, and socio-environmental factors still need to be singled out for detailed discussion, even though relevant sub-factors are involved, mainly due to the following considerations. Firstly, despite the importance of the political factors, this study focuses on the factors that have a more significant direct impact on the regeneration of brownfield sites and have a more substantial operative effect, which directly affects the physical and socio-economic aspects of brownfield regeneration.

Moreover, they require different analytical frameworks and involve a more comprehensive range of external variables, which may add to the complexity of the study. Secondly, economic factors usually require consideration of broader economic trends and market dynamics. However, due to the local nature of the study, the brownfield regeneration strategy in Xi’an, which did not analyze the broader economic conditions in detail, was focused on the actual and local situation. Therefore, the direct economic conditions and market dynamics factors are relatively more important in the assessment system. Thirdly, the importance of remediation technologies for brownfield landscape regeneration is undisputed. However, most of today’s brownfield research is centered around remediation technologies. There are already a large number of existing conditions and practical strategies that can be implemented using existing technologies, and the inclusion of a technology factor would require an exploration of future technological developments, but this would introduce significant uncertainties and assumptions that could diminish the direct applicability and relevance of the study. Therefore, including technology as a separate factor has yet to be explicitly addressed. Explicitly include technology as a separate factor. Fourth, the social-environmental factors cover community participation, social capital, and environmental testing. However, this research focuses on environmental remediation needs and sustainable development practices at brownfield sites in Xi’an. In contrast, the socio-environmental factors require detailed quantitative measurements and data analysis over a long and ongoing basis beyond this research’s timeframe and resource constraints. It was, therefore, not included as a separate factor but is represented explicitly in the Level D subfactors.

Although the above factors were not addressed separately in detail in this study due to practical considerations related to the scope of the study, resource constraints, and direct applicability of the findings, future research could start with these factors to provide a more comprehensive and multidimensional understanding of brownfield regeneration, mainly through longitudinal data studies and broader stakeholder engagement.

### 4.2 Comparison of similar research

The assessment results show that physical factors are most important in brownfield landscape regeneration, as functional and cultural factors and emotional and informational factors. By comparing this research results with similar studies in regions such as Europe and North America, the uniqueness of the historical and cultural factors and the common factors of physical, functional, and emotional factors in Xi’an can be found. First, cultural factors play a crucial role in the regeneration of brownfield landscapes in Xi’an, China, where studies have shown that the preservation and enhancement of cultural heritage not only maintains the cultural identity and historical continuity of the community but also promotes tourism. Studies of urban brownfield sites such as Boğazkale in Turkey and Kyoto in Japan, for example, similarly emphasize historical culture as an essential aspect of urban brownfield regeneration and recognize the dual benefits of cultural preservation and tourism enhancement. These studies show the generalized importance of cultural factors in different parts of the globe [83,84].

Secondly, the physical factors emphasize the importance of infrastructure improvements, environmental remediation, and aesthetic enhancement of brownfield sites, which are vital factors common to all successful brownfield regeneration projects. Studies conducted in Xi’an and other cities in North America and Europe, such as studies conducted by scholars in Detroit in the United States and Manchester in the United Kingdom, have emphasized similar improvements in physical factors [85,86]. These studies emphasize the global recognition of infrastructure and environmental factors as critical to successful brownfield regeneration. This research suggest that by improving infrastructure, adding public facilities, and upgrading environmental quality, Xi’an’s project not only improves the living conditions of its residents but also enhances the region’s economic attractiveness.

In addition, functional factors are an important common denominator. Functional factors focus on brownfield regeneration spaces’ availability, adaptive reuse, and flexibility. For example, Russo A study, in line with these findings, promotes multifunctional spaces and adaptive reuse to accommodate changing urban needs. This study also emphasizes the importance of such adaptability and flexibility to achieve greater sustainability and cost-effectiveness by reusing structures and facilities in existing brownfield spaces [87].

Regarding emotional factors in the regeneration of brownfield landscapes, the research found that residents’ sense of belonging and community pride in communities surrounding brownfield sites can be significantly enhanced by creating brownfield regeneration spaces where residents feel a sense of belonging and pride. Studies by United States and Italy scholars have also confirmed the importance of emotional and psychological factors in revitalizing urban brownfield sites, emphasizing the need to create spaces that enhance the well-being of residents and the spirit of community [88,89].

Finally, information factors are an integral part of development in the current information age, and studies across the globe have demonstrated the importance of data-driven decision-making, transparency, and brownfield management improvements. Intelligent technologies and data analytics to guide urban brownfield planning and regeneration projects have become a trend in brownfield studies in London, United Kingdom, and Emilia-Romagna, Italy [90,91]. This research emphasizes the timeliness and accuracy of online and offline information to ensure the science of brownfield landscape regeneration management.

### 4.3 AHP method matching and related dominant factors

The AHP assessment model of the B, C, and D level assessment indexes presents a sequential and gradually clear relationship. In this research, based on five brownfield types in Xi’an, 20 experts with landscape and architecture backgrounds were invited to assess the dominant factors of brownfield landscape regeneration in Xi’an. Despite the differences in professional perspectives and the different ordering of the importance of physical and functional factors, a comprehensive analysis shows that the assessment results are statistically consistent with the selection logic. Therefore, this research starts from the assessment results of Level 1 Factor B, connects the dominant specific indicators in the assessment factors C and D, and derives the potential dominant factors for the regeneration of five types of brownfield landscapes in Xi’an, which will lead to the discussion of the main factors for the regeneration of brownfield sites in Xi’an.

In this research, a review of the brownfield governance literature reveals that many studies have only explored a particular factor in-depth and have mainly discussed the current status of soil contamination and governance methods centered on functional factors of brownfields. However, in this research, the physical factor layer was dominated by a comprehensive assessment by 20 experts. Although functional factors are crucial, based on the current brownfield status and remediation techniques, brownfield landscape regeneration has been transformed from a technical challenge to an exploration of sustainability and cultural diversity inheritance. Therefore, the current brownfield management should pay more attention to excavating the characteristics and connotations of the existing objects and factors of brownfield sites and maximize the retention of the physical attributes of the sites. Therefore, in brownfield landscape regeneration, focusing on the landscape’s spatial features and visual-physical factors of character and feature excavation is necessary.

Of course, based on the differences between the five different brownfield types selected, planners are required to take complete account of the uniqueness of each kind of brownfield site when formulating regeneration strategies and developing targeted governance and development programs. For example, industrial and transportation brownfield sites are usually located in the urban core or transportation hubs and have high redevelopment potential. However, issues of pollution and infrastructure improvement need to be addressed. On the other hand, mining brownfields and landfills face more complex ecological and environmental governance challenges. They are more expensive to develop but can also realize ecological and economic benefits through effective governance measures. Military brownfields require special attention to identifying safety hazards and land use conversion due to their particular historical uses. Therefore, based on this study’s assessment of the potential dominant factors of brownfield sites in Xi’an, an in-depth analysis of the differences between these different types of brownfield sites is also needed to provide a more scientific and comprehensive guide for urban brownfield regeneration.

### 4.4 Application case study

**Fig 7:**
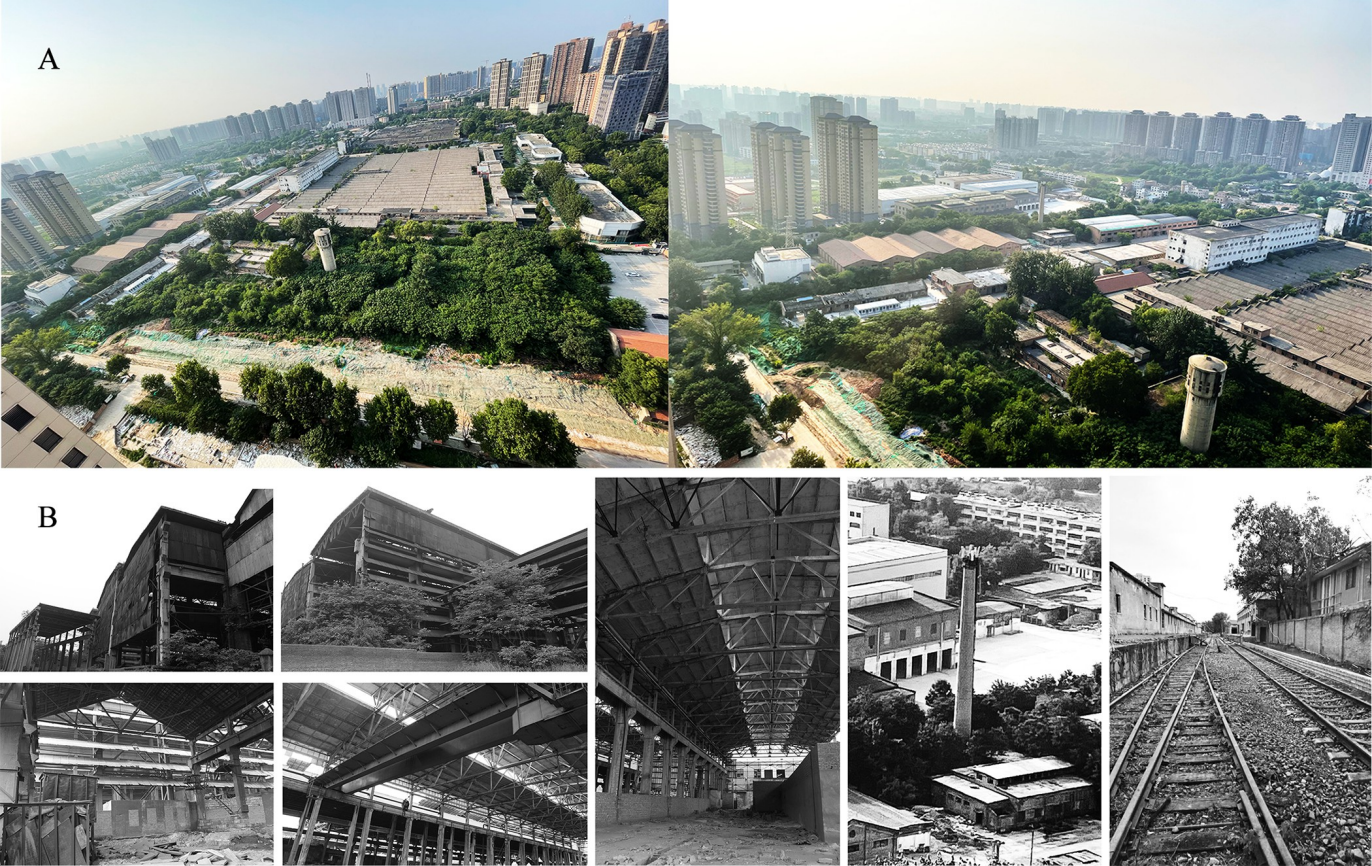
Site spatial-physical status and industrial heritage construction. Image A represents an aerial view of the target site, which provides a specific understanding of the site’s internal environment, remaining structures, and vegetative cover. Image B shows the physical and cultural elements of the brownfield site, such as the spatial setting, historic industrial buildings on the site, and abandoned railroad tracks. These elements provide the basis for implementing specific brownfield landscape regeneration strategies. The copyright of the figures is available in S1 Appendix.

**Fig 8:**
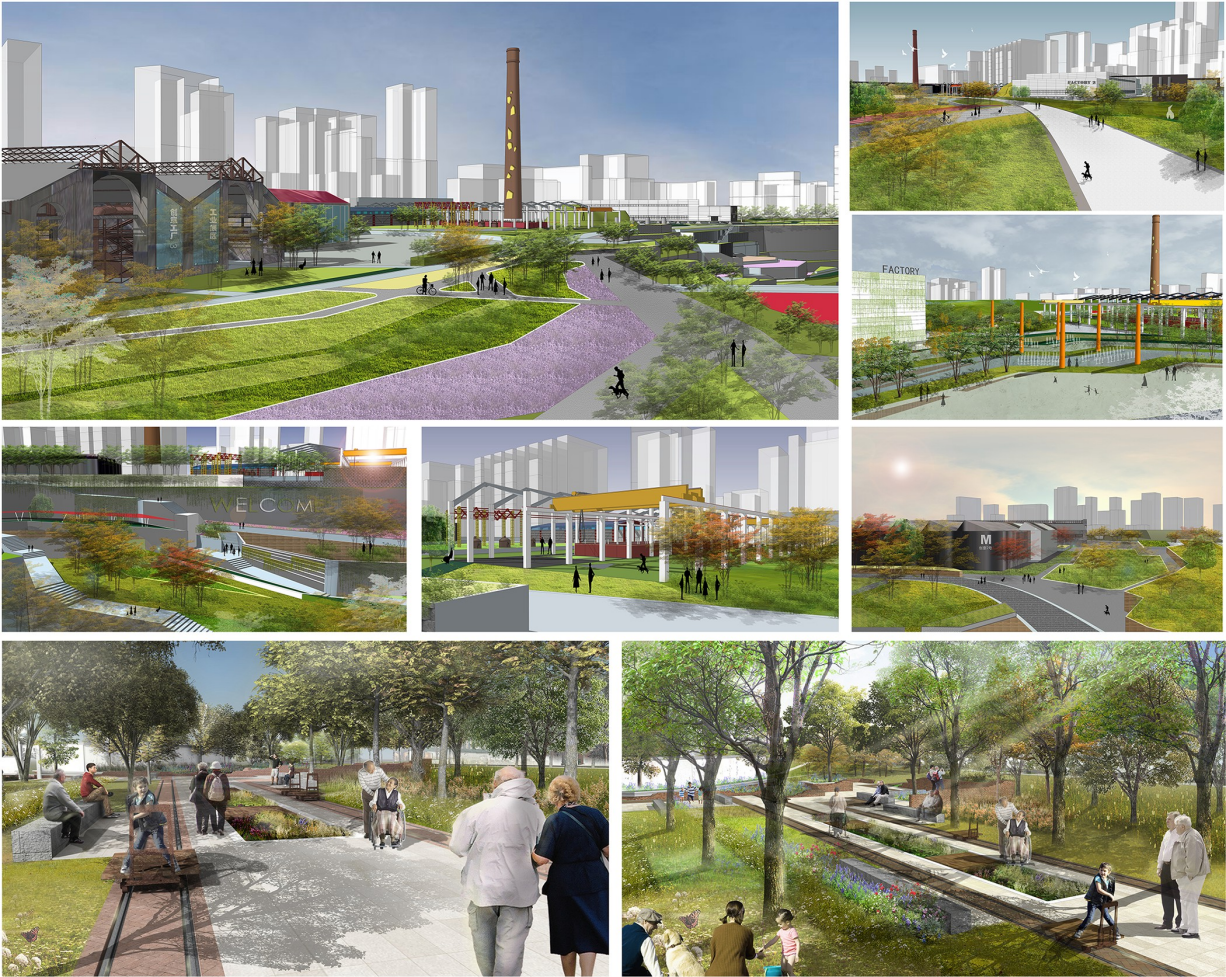
Landscape regeneration of industrial buildings, structures, terrain, and plants. The figures show that based on the spatial characteristics of the brownfield site, the physical elements of the site, such as abandoned factories, railroad tracks, and chimneys, are renewed with the language of landscape design. The design practice of brownfield landscape regeneration is based on the functional factors of the surrounding area’s public interaction, leisure, and educational needs. To activate the regeneration of the brownfield site and the surrounding environment while highlighting the culture of the industrial site. The source and copyright of the figures are available in S1 Appendix.

To more intuitively reflect the assessed potential dominant factors in the landscape regeneration of brownfield sites in Xi’an, the specific case was selected as the most representative industrial brownfield among the five brownfield types in Xi’an in this study, i.e., in the Northwest National Cotton Factory No. 3, to carry out the landscape regeneration and renovation intention design. The Northwest State Cotton Factory is located in the textile city southwest of Baqiao District in Xi’an, China. It is the northwest region’s most extensive textile industrial base, as shown in Fig 1. The factory was built in 1980, closed in 2008, and has been abandoned since, with an area of approximately 202,920 square meters, as shown in Fig 7. By digging deeper into the characteristics and features of these physical factors, the site’s historical, cultural, and ecological values can be better understood, providing a more comprehensive and sustainable solution for brownfield landscape regeneration, as shown in Fig 8.

However, functionality remains an important issue that every brownfield landscape regeneration must face and address. Brownfield landscape regeneration is essentially the restoration of the landscape ecosystem of the site, so while improving the comfort of the site environment, it is necessary to ensure basic site safety and functional convenience. Among them, the safety of pollutants is crucial, especially for treating heavy metal pollution in soil and water. The most effective method is the guest soil and plant adsorption methods, as shown in Fig 7. The physisorption method is widely used to purify the soil and water by absorbing different heavy metals from various plants, requiring a certain degree of plant green coverage. The purpose of urban brownfield landscape regeneration is to make up for the lack of urban space and function and to meet the different needs of the public. Therefore, brownfield landscape regeneration must start from the needs of the public around the site. Brownfield landscape regeneration is a long-cycle process; in the early stage of landscape regeneration, it mainly solves the main problems of Brownfield; informational factors and emotional factors can be implanted in the later stage of landscape regeneration in the functional blocks.

To summarize, brownfield sites, as historical remains in urban development, have witnessed the historical changes of cities and have irreplaceable historical and cultural value. In contemporary society, sustainable development requires the sustainability of economic growth and emphasizes the inheritance and continuation of cultural genes. Therefore, in the process of brownfield landscape regeneration, the historical and cultural connotations of brownfield sites must be fully excavated and demonstrated, especially for cultural capitals like Xi’an, which has a history of 13 dynasties; it is more important to pay attention to the diversity of historical and cultural manifestations and the accuracy of the expressions in the process of brownfield landscape regeneration in different regions. At the same time, it is also necessary to actively integrate modern elements to enhance the richness and artistry of the landscape and reflect the contemporary characteristics of cultural heritage. Brownfield landscape regeneration is a long-term process, with the initial stages primarily focused on addressing the primary issues of brownfields. Information and emotional factors can be incorporated into functional components during the later stages of landscape regeneration.

### 4.5 Research limitations and prospects

This research takes Xi’an, a famous historical and cultural city in China, as an object and adopts the AHP to construct an adaptive assessment system, explore the dominant factors of brownfield landscape regeneration, formulate a specific guiding strategy, and provide practical suggestions for urban planning and regeneration. However, some directions and those that need to be further optimized in the future are worth discussing.

First, the assessment framework relies on expert assessment, which may introduce subjective bias despite efforts to ensure rigor and consistency. Although 20 experts were selected based on the range of academic choices for the sample size of the AHP method, the findings may need more broad applicability and reliability due to the small sample size. Future studies should expand the sample size to include experts from different disciplines, regions, and stakeholders to mitigate potential bias and improve objectivity through continuous environmental monitoring and quantitative measurement. Secondly, this research comprehensively reviewed the literature and consulted with experts to cover the main factors mentioned in academic discussions and confirmed the relevance of these factors through expert assessment. This research mainly focuses on the selection and evaluation of factors at a macro level based on the perspectives of design and landscape studies and focuses on the discussion of the dominant factors and assessment of urban brownfield sites in Xi’an, China; there may be some factors that are not emphasized as well as elaborated on, for example, political, economic, technological, and legal factors. Future research could ensure a more detailed assessment of all potential factors by utilizing a comprehensive PESTEL (Political, Economic, Social, Technological, Environmental, and Legal) analysis.

Furthermore, this research proposes a flexible and adaptable assessment framework based on the AHP methodology in the context of urban brownfield sites in Xi’an, China. Although the specific strategies developed are based on the unique context of Xi’an, they are generalizable for Chinese cities and suitable for most brownfield landscape regeneration plans in China. However, they may not directly apply to cities and countries with different social, economic, cultural, and environmental characteristics. The research uses Xi’an City as a case study and is limited to a few common types of brownfield sites, and the results will be constrained by the sites selected and the data available. Future research can be based on the framework system of this assessment and flexibly adapted in different regional and national socio-economic and cultural contexts to incorporate more brownfield factors, such as economic, social, cultural, and policy, to validate and extend this research. In addition, collecting quantitative and longitudinal data, such as conducting long-term environmental detection tracking studies, regularly collecting environmental, social, and economic data after implementing assessment projects, and observing how these factors change over time, ensures the study’s objectivity. Comparative studies are conducted across multiple cities to identify common factors and differences, thereby increasing the robustness and generalizability of the findings. Finally, this research is a staged exploration that must capture long-term trends or future developments. Future studies could explore this, including detailed implementation plans that address practical challenges, resource needs, timelines, and potential barriers. In the meantime, conducting case studies and pilot projects can provide valuable insights into practical applications that can help further refine strategies and develop more comprehensive and feasible tactics. Despite these limitations, this research hopes to make valuable practical and academic contributions to understanding brownfield regeneration dynamics and applying the AHP methodology to the sustainable development of urban brownfields, using the city of Xi’an, China, as a base for exploration and analysis.

## 5. Conclusions

Brownfield landscape regeneration is a complex and lengthy process that requires consideration of many factors. Targeted analysis of the dominant factors of different brownfield types and prioritized, step-by-step planning are effective methods for brownfield landscape regeneration. At the same time, since China has not yet established a brownfield database and a systematic evaluation system for brownfield development due to the late start of brownfield research in China, this study takes Xi’an, China, as an example and explores the establishment of a reasonable evaluation system, which is crucial for the planning and management of brownfield landscape regeneration in Xi’an and even in China.

This research used the AHP to summarize and disassemble the complex problems of five types of brownfield sites in Xi’an, and a three-level assessment factor matrix was constructed. The main factors were selected layer by layer according to the weight order based on the current situation data collection and expert assessment. Finally, the dominant factors for landscape regeneration of the five brownfield sites in Xi’an were comprehensively assessed. However, this assessment system still has some limitations and needs to be tested through the planning in brownfield landscape regeneration to check its rationality. However, the significance of this research lies in the fact that the established adaptable assessment framework and methodology can be flexibly adjusted according to the economic and cultural contexts of different regions as well as cities, which provides new perspectives for the formation and improvement of assessment systems for brownfield landscape regeneration in other countries and territories. At the same time, practical solutions to brownfield landscape regeneration challenges by comparing various types of brownfield sites across multiple cities, identifying common factors and differences, incorporating more public and stakeholder views, reducing potential bias, increasing the comprehensiveness of the research, and developing a detailed landscape regeneration implementation plan are important aspects of future brownfield development research.

## Supporting information

S1 Appendix Figure analyzing maps source and copyright statement. Relevant studies analyzed the map source of the figure and redesigned it based on the original base map. Involves on-site photographs taken during the research and a copyright statement for the design drawings.
(DOCX)

S1 Raw images (PDF)

## References

[1] Adams D, De Sousa C, Tiesdell S. Brownfield Development: A Comparison of North American and British Approaches. Urban studies. 2009;47: 75–104. doi: 10.1177/0042098009346868

[2] Liebmann H, Kuder T. Pathways and Strategies of Urban Regeneration—Deindustrialized Cities in Eastern Germany. European Planning Studies. 2012;20: 1155–1172. doi: 10.1080/09654313.2012.674348

[3] Nassauer JI, Raskin J. Urban Vacancy and Land Use Legacies: A Frontier for Urban Ecological Research, Design, and Planning. Landscape and urban planning. 2014;125: 245–253. doi: 10.1016/j.landurbplan.2013.10.008

[4] Andersson-Sköld Y, Klingberg J, Gunnarsson B, Cullinane K, Gustafsson I, Hedblom M, et al. A Framework for Assessing Urban Greenery’s Effects and Valuing Its Ecosystem Services. Journal of Environmental management. 2018;205: 274–285. doi: 10.1016/j.jenvman.2017.09.071 29020655

[5] Alker S, Joy V, Roberts P, Smith N. The Definition of Brownfield. Journal of Environmental Planning and Management. 2000;43: 49–69. doi: 10.1080/09640560010766

[6] De Sousa C.A. Unearthing the benefits of brownfield to green space projects: An examination of project use and quality of life impacts. Local Environment. 2006;11(5): 577–600. doi: 10.1080/13549830600853510

[7] Huang L, Zheng W, Hong J, Liu Y, Liu G. Paths and strategies for sustainable urban renewal at the neighbourhood level: A framework for decision-making. Sustainable Cities and Society. 2020;55: 102074. doi: 10.1016/j.scs.2020.102074

[8] Dixon T, Pocock Y, Waters M. An Analysis of the UK Development Industry’s Role in Brownfield Regeneration. Journal of Property Investment & Finance. 2006;24: 521–541. doi: 10.1108/14635780610708310

[9] Hayek M, Novak M, Arku G, Gilliland J. Mapping Industrial Legacies: Building a Comprehensive Brownfield Database in Geographic Information Systems. Planning Practice & Research. 2010;25: 461–475. doi: 10.1080/02697459.2010.511018

[10] Ruelle C, Halleux JM, Teller J. Landscape Quality and Brownfield Regeneration: A Community Investigation Approach Inspired by Landscape Preference Studies. Landscape Research. 2013;38: 75–99. doi: 10.1080/01426397.2011.647898

[11] Loures L, Panagopoulos T, Burley JB. Assessing User Preferences on Post-Industrial Redevelopment. Environment and Planning B: Planning and Design. 2016;43: 871–892. doi: 10.1177/0265813515599981

[12] Nocca F, Fusco Girard L. Towards an Integrated Assessment Approach for Cultural Urban Landscape Conservation/Regeneration. Region. 2018;5: 33–51. doi: 10.18335/region.v5i1.160

[13] Shojaee-Far M. Assessment of Abandoned Properties in Geopolitical Conflict Zones. Journal of European Real Estate Research. 2021;15:130–144. doi: 10.1108/jerer-03-2020-0016

[14] Otsuka N, Dixon T, Abe H. Stock Measurement and Regeneration Policy Approaches to ’Hardcore’ Brownfield Sites: England and Japan Compared. Land Use Policy. 2013;33: 36–41. doi: 10.1016/j.landusepol.2012.12.002

[15] Cui Y, Fang W. Landscape Design Methodology of Sustainable Brownfield Regeneration. In 2015 International Conference on Industrial Technology and Management Science; 2015 March 27-28; Tianjin, China. Atlantis Press; 2015. p. 1452–1454. doi: 10.2991/itms-15.2015.354

[16] Zheng X, Kirkwood NG. Landscape architecture and sustainable remediation. In: Hou D, editor. Sustainable Remediation of Contaminated Soil and Groundwater. 1st ed. Oxford, United Kingdom: Butterworth-Heinemann; 2020. pp. 301–324. doi: 10.1016/B978-0-12-817982-6.00012-4

[17] Mejia MP, Rojas CA, Curd E, Renshaw MA, Edalati K, Shih B, et al. Soil Microbial Community Composition and Tolerance to Contaminants in an Urban Brownfield Site. Microbial ecology. 2022;85: 998–1012. doi: 10.1007/s00248-022-02061-1 35802172 PMC10156844

[18] Shen X, Ge M, Handel SN, Wang W, Jin Z, Kirkwood NG. Advancing Environmental Design with Phytoremediation of Brownfield Soils Using Spontaneous Invasive Plants. Science of The Total Environment. 2023;883: 163635. doi: 10.1016/j.scitotenv.2023.163635 37100146

[19] Fernández-Braña A, Salgado L, Gallego JLR, Afif E, Boente C, Forján R. Phytoremediation Potential Depends on the Degree of Soil Pollution: A Case Study in an Urban Brownfield. Environmental Science and Pollution Research. 2023;30: 67708–67719. doi: 10.1007/s11356-023-26968-5 37118389 PMC10203031

[20] De Sousa C. Brownfield Redevelopment versus Greenfield Development: A Private Sector Perspective on the Costs and Risks Associated with Brownfield Redevelopment in the Greater Toronto Area. Journal of environmental planning and management. 2000;43: 831–853. doi: 10.1080/09640560020001719

[21] Thornton G, Franz M, Edwards D, Pahlen G, Nathanail P. The Challenge of Sustainability: Incentives for Brownfield Regeneration in Europe. Environmental science & policy. 2007;10: 116–134. doi: 10.1016/j.envsci.2006.08.008

[22] Wedding GC, Crawford-Brown D. Measuring Site-Level Success in Brownfield Redevelopments: A Focus on Sustainability and Green Building. Journal of environmental management. 2007;85: 483–495. doi: 10.1016/j.jenvman.2006.10.018 17240521

[23] Williams K, Dair C. A Framework for Assessing the Sustainability of Brownfield Developments. Journal of Environmental Planning and Management. 2007;50: 23–40. doi: 10.1080/09640560601048275

[24] Adams D, De Sousa C, Tiesdell S. Brownfield Development: A Comparison of North American and British Approaches. Urban studies. 2009;47: 75–104. doi: 10.1177/0042098009346868

[25] Schädler S, Morio M, Bartke S, Rohr-Zänker R, Finkel M. Designing Sustainable and Economically Attractive Brownfield Revitalization Options Using an Integrated Assessment Model. Journal of Environmental Management. 2011;92: 827–837. doi: 10.1016/j.jenvman.2010.10.026 21051134

[26] Aronson J, Goodwin N, Orlando L, Eisenberg C, Cross AT. A World of Possibilities: Six Restoration Strategies to Support the United Nation’s Decade on Ecosystem Restoration. Restoration Ecology. 2020;28: 730–736. doi: 10.1111/rec.13170

[27] Rusciano V, Civero G, Scarpato D. Social and Ecological High Influential Factors in Community Gardens Innovation: An Empirical Survey in Italy. Sustainability. 2020;12: 4651. doi: 10.3390/su12114651

[28] Mahammedi C, Mahdjoubi L, Booth C, Bowman R, Butt TE. Criteria for Preliminary Risk Assessment of Brownfield Site: An International Survey of Experts. Environmental Management. 2022;70: 681–696. doi: 10.1007/s00267-022-01684-x 35925209 PMC9439978

[29] Palm E, Guidi Nissim W, Gagnon-Fee D, Labrecque M. Photosynthetic Patterns during Autumn in Three Different Salix Cultivars Grown on a Brownfield Site. Photosynthesis Research. 2022;154: 155–167. doi: 10.1007/s11120-022-00958-z 36104474 PMC9630210

[30] Hammond EB, Coulon F, Hallett SH, Thomas R, Hardy D, Beriro DJ. Digital Tools for Brownfield Redevelopment: Stakeholder Perspectives and Opportunities. Journal of Environmental Management. 2023;325: 116393. doi: 10.1016/j.jenvman.2022.116393 36270126

[31] Jiang Z, Shi Y, Chen X, Xu G, Lv S. Research on the Evolution and Influencing Factors of Public Preferences of Brownfield Land Green Space Conversion in China. International Journal of Environmental Research and Public Health. 2023;20: 1274. doi: 10.3390/ijerph20021274 36674028 PMC9859313

[32] Loures L, Panagopoulos T. Sustainable reclamation of industrial areas in urban landscapes. Available from: http://hdl.handle.net/10400.1/12122. Accessed in 2007. doi: 10.2495/SDP070752

[33] Krejčí T, Martinat S, Klusacek P, Tochackova K. Brownfields and Tourism Development—Visitors preferences exampled on Brno City. In: Zitek V, editors. In Proceedings of the 17th International colloquium on regional sciences; 2014. pp. 857–865. doi: 10.5817/CZ.MUNI.P210-6840-2014-111

[34] Mathey J, Arndt T, Banse J, Rink D. Public Perception of Spontaneous Vegetation on Brownfields in Urban Areas—Results from Surveys in Dresden and Leipzig (Germany). Urban Forestry & Urban Greening. 2018;29: 384–392. doi: 10.1016/j.ufug.2016.10.007

[35] Yagci E, Nunes da Silva F. The Future of Post-Industrial Landscapes in East Lisbon: The Braço de Prata Neighbourhood. Sustainability. 2021;13: 4461. doi: 10.3390/su13084461

[36] Lin H, Zhu Y, Zhou J, Mu B, Liu C. Understanding stakeholder relationships in sustainable brownfield regeneration: A combined FAHP and SNA approach. Environment, Development and Sustainability. 2024;26: 15823–15859. doi: 10.1007/s10668-023-03275-0

[37] Xi S. Transforming urban industrial wastelands using a CNN-based land classification model. Soft Computing. 2024;28: 1317–1335. doi: 10.1007/s00500-023-09458-1

[38] Swensen G, Granberg M. The Impact of Images on the Adaptive Reuse of Post-Industrial Sites. The Historic Environment: Policy & Practice. 2024;15: 101–129. doi: 10.1080/17567505.2024.2311005

[39] Krejčí T, Dostál I, Havlíček M, Martinát S. Exploring the Hidden Potential of Sugar Beet Industry Brownfields (Case Study of the Czech Republic). Transportation Research Part D: Transport and Environment. 2016;46: 284–297. doi: 10.1016/j.trd.2016.04.006

[40] Abdullahi S, Pradhan B. Sustainable Brownfields Land Use Change Modeling Using GIS-Based Weights-of-Evidence Approach. Applied spatial analysis and policy. 2016;9: 21–38. doi: 10.1007/s12061-015-9139-1

[41] Abdullahi S. Brownfield land use change modeling using GIS-based Weights-of-Evidence approach. Journal of Radar and Optical Remote Sensing and GIS. 2021;4: 66–77.

[42] Oudes D, Stremke S. Climate Adaptation, Urban Regeneration, and Brownfield Reclamation: A Literature Review on Landscape Quality in Large-Scale Transformation Projects. Landscape Research. 2020;45: 905–919. doi: 10.1080/01426397.2020.1736995

[43] Mastervich B, Garbach K, Harwell MC. Enhancing multiple benefits of brownfield cleanups by applying ecosystem services concepts. Frontiers in Ecology and Evolution. 2024;12: 1286150. doi: 10.3389/fevo.2024.1286150 PMC10936587 38487592

[44] Szabó M, Bozsoki F. Redevelopment of Brownfields for Cultural Use from ERDF Fund—The Case of Hungary between 2014 and 2020. Journal of Risk and Financial Management. 2022;15: 181. doi: 10.3390/jrfm15040181

[45] Turečková K, Nevima J, Duda D, Tuleja P. Latent Structures of Brownfield Regeneration: A Case Study of Regions of the Czech Republic. Journal of Cleaner Production. 2021;311: 127478. doi: 10.1016/j.jclepro.2021.127478

[46] Wan Y, Chen S, Liu J, Liu J, Jin L. Brownfield-related studies in the context of climate change: A comprehensive review and future prospects. Heliyon. 2024. doi: 10.1016/j.heliyon.2024.e25784 38420456 PMC10900957

[47] Dong W, Lin G. Integrated Decision-Making of Urban Agriculture within the Greyfield Regeneration Environments (UAGR). Buildings. 2024;14: 1415. doi: 10.3390/buildings14051415

[48] Ansenberg U, Marom N. The Multiple Enactments of Contamination: Rethinking the Remediation and Redevelopment of Military‐Industrial Brownfields in the Tel Aviv Region. International Journal of Urban and Regional Research. 2024;48: 7–30. doi: 10.1111/1468-2427.13220

[49] Zhu H. Study on Landscape Ecological Restoration and Optimization Design of Abandoned Mining Area—Take Pingdingshan Seven Mine as an Example. M.Sc. Thesis, Xi’an University of Architecture and Technology. 2019. Available from: https://chn.oversea.cnki.net/KCMS/detail/detail.aspx?dbcode=CMFD&dbname=CMFD202001&filename=1020616185.nh&uniplatform=OVERSEA&v=Rkiw9s3ktKuzWsuJ_hFZhHis3vtpx_K54ryZxFLzOe5izFjkMlqZ5ihyFv2Glg16.

[50] Feng S, Hou W, Chang J. Changing Coal Mining Brownfields into Green Infrastructure Based on Ecological Potential Assessment in Xuzhou, Eastern China. Sustainability. 2019;11: 2252. doi: 10.3390/su11082252

[51] Zhong Q, Zhang L, Zhu Y, Konijnendijk van den Bosch C, Han J, Zhang G, et al. A Conceptual Framework for Ex Ante Valuation of Ecosystem Services of Brownfield Greening from a Systematic Perspective. Ecosystem Health and Sustainability. 2020;6. doi: 10.1080/20964129.2020.1743206

[52] Jin X, Qian S, Yuan J. Identifying urban rewilding opportunity spaces in a metropolis: Chongqing as an example. Ecological Indicators. 2024;160: 111778. doi: 10.1016/j.ecolind.2024.111778

[53] Vaz E. Artificial terrariums as urban habitats innovative solutions for environmental, economic, and social development. Habitat International. 2024;144: 103017. doi: 10.1016/j.habitatint.2024.103017

[54] Zhu C, Zhang H. Research on the Influence of Brownfield Railway Space on the Change of Human Psychological Perception, Based on Analysis of Gestalt Psychology and Environmental Psychology. Art and Design. 2021;7: 52–54. doi: 10.16824/j.cnki.issn10082832.2021.07.010

[55] Song Y, Lyu Y, Qian S, Zhang X, Lin H, Wang S. Identifying Urban Candidate Brownfield Sites Using Multi-Source Data: The Case of Changchun City, China. Land Use Policy. 2022;117: 106084. doi: 10.1016/j.landusepol.2022.106084

[56] Wang Y, Li Z, Zheng X. The Microclimatic Effects of Ecological Restoration in Brownfield Based on Remote Sensing Monitoring: The Case Studies of Landfills in China. Ecological Engineering. 2020;157: 105997. doi: 10.1016/j.ecoleng.2020.105997

[57] Radivojević G, Gajović V. Supply chain risk modeling by AHP and Fuzzy AHP methods. Journal of Risk Research. 2014;17: 337–352. doi: 10.1080/13669877.2013.808689

[58] Naghadehi MZ, Mikaeil R, Ataei M. The application of fuzzy analytic hierarchy process (FAHP) approach to selection of optimum underground mining method for Jajarm Bauxite Mine, Iran. Expert systems with applications. 2009;36: 8218–8226. doi: 10.1016/j.eswa.2008.10.006

[59] Zhang J, Qu Q, Chen XB. Assessing the sustainable safety practices based on human behavior factors: An application to Chinese petrochemical industry. Environmental Science and Pollution Research. 2022;29: 44618–44637. doi: 10.1007/s11356-022-18909-5 35133599

[60] Qingyou Y, Guangyu Q, Qifeng W, Yang Y, Yunfeng Z. Research on the construction and application strategy of precision marketing model for industrial and commercial customers. In: IOP conference series: earth and environmental science; 2021 July 9-11; Chengdu, China. IOP Publishing; 2021. p. 012037. doi: 10.1088/1755-1315/831/1/012037

[61] Gong Q, Chen BZ, Zhi PP, Li YH. Comprehensive evaluation for design for design scheme of metro auxiliary power supply system based on FAHP. IOP Conference Series: Materials Science and Engineering; 2021 October 8th-11th; Shaanxi, China. IOP Publishing; 2021. p. 022014. doi: 10.1088/1757-899X/1043/2/022014

[62] Hamdia KM, Arafa M, Alqedra M. Structural damage assessment criteria for reinforced concrete buildings by using a Fuzzy Analytic Hierarchy process. Underground Space. 2018;3: 243–249. doi: 10.1016/j.undsp.2018.04.002

[63] Yariyan P, Zabihi H, Wolf ID, Karam M, Amiriyan S. Earthquake risk assessment using an integrated Fuzzy Analytic Hierarchy Process with Artificial Neural Networks based on GIS: A case study of Sanandaj in Iran. International Journal of Disaster Risk Reduction. 2020;50: 101705. doi: 10.1016/j.ijdrr.2020.101705

[64] Lee HS, Park EY. Developing a Landscape Sustainability Assessment Model Using an Analytic Hierarchy Process in Korea. Sustainability. 2019;12: 301. doi: 10.3390/su12010301

[65] Feng SJ, Chao NC, Chen YJ. Taiwan Brownfield Redevelopment & Ecological Restoration Indicator Analysis. In IOP Conference Series: Earth and Environmental Science; 2019 January 26–29; Seoul, South Korea. IOP Publishing; 2019. p. 012017. doi: 10.1088/1755-1315/291/1/012017

[66] Feng SJ, Tung CE. A Study of Ecological Restoration Indicators of the Brownfields of Shuinandong, Taiwan. 10th Int Conf Future Environ Energy; 2020 January 7-9; Kyoto, Japan. IOP Publishing; 2020. p. 012043. doi: 10.1088/1755-1315/581/1/012043

[67] Lin Y, Wu B, Wang D, Xiao W, Huang Y, Fu S, et al. Comprehensive assessment of Ecosystem Services for Brownfield Redevelopment in Changsha. Arabian Journal of Geosciences. 2021;14: 1021. doi: 10.1007/s12517-021-07261-6

[68] Cappai F, Forgues D, Glaus M. Methodological Approach for Evaluating Brownfield Redevelopment Projects. Urban Science. 2019;3: 45. doi: 10.3390/urbansci3020045

[69] Zou J. Study on Landscape Spatial quality Evaluation of Brownfield Regeneration in Shanghai. M.Sc. Thesis, East China Normal University. 2019. Available from: https://kns.cnki.net/KCMS/detail/detail.aspx?dbname=CMFD201902&filename=1019834890.nh.

[70] Milošević DM, Milošević MR, Simjanović DJ. Implementation of adjusted fuzzy AHP method in the assessment for reuse of industrial buildings. Mathematics. 2020;8: 1697. doi: 10.3390/math8101697

[71] Ding R, Yu K, Fan Z, Liu J. Study and Application of Urban Aquatic Ecosystem Health Assessment Index System in River Network Plain Area. International Journal of Environmental Research and Public Health. 2022;19: 16545. doi: 10.3390/ijerph192416545 36554433 PMC9779142

[72] Xiang Z, Zhou T, Chen J. Fuzzy synthetic assessment model for ecological security risks of water reservoirs based on AHP-entropy weight method and its application. In: Int Conf Sustain Technol Manag (ICSTM 2022); 2022 JULY 22-24; Macao, China. SPIE; 2022. p. 244–249. doi: 10.1117/12.2644691

[73] Wang L, Yao J, Zhang H, Pang Q, Fang M. A sustainable shipping management framework in the marine environment: Institutional pressure, eco-design, and cross-functional perspectives. Frontiers in Marine Science. 2023;9: 1070078. doi: 10.3389/fmars.2022.1070078

[74] Meniz B, Özkan EM. Vaccine selection for COVID-19 by AHP and novel VIKOR hybrid approach with interval type-2 fuzzy sets. Engineering applications of artificial intelligence. 2023;119: 105812. doi: 10.1016/j.engappai.2022.105812 36624893 PMC9812846

[75] Yadav R, Lee HH. Ranking and selection of dental restorative composite materials using FAHP-FTOPSIS technique: An application of multi criteria decision making technique. ournal of the Mechanical Behavior of Biomedical Materials. 2022;132: 105298. doi: 10.1016/j.jmbbm.2022.105298 35660553

[76] Guru S, Verma S, Baheti P, Dagar V. Assessing the feasibility of hyperlocal delivery model as an effective distribution channel. Management Decision. 2023;61: 1634–1655. doi: 10.1108/MD-03-2022-0407

[77] Naveed QN, Qahmash AI, Qureshi MRN, Ahmad N, Abdul Rasheed MA, Akhtaruzzaman M. Analyzing Critical Success Factors for Sustainable Cloud-Based Mobile Learning (CBML) in Crisp and Fuzzy Environment. Sustainability. 2023;15: 1017. doi: 10.3390/su15021017

[78] Lin HH. Application of a fuzzy decision model to the design of a pillbox for medical treatment of chronic diseases. Applied Sciences. 2019;9: 4909. doi: 10.3390/app9224909

[79] Lin HH, Cheng JH. Application of Fuzzy Decision Model Selection of Product in Human Factors Design. In: Human Aspects of IT for the Aged Population. Healthy and Active Aging: 6th International Conference, ITAP 2020, Held as Part of the 22nd HCI International Conference, HCII 2020: Proceedings Part II 22; 2020 July 19–24; Copenhagen, Denmark. Springer International Publishing; 2020. p. 101-112. doi: 10.1007/978-3-030-50249-2_8

[80] Wu HC, Chen TCT, Huang CH, Shih YC. Comparing built-in power banks for a smart backpack design using an auto-weighting fuzzy-weighted-intersection FAHP approach. Mathematics. 2020;8: 1759. doi: 10.3390/math8101759

[81] Liu H, Guo C, Zhang B. Attractiveness consumption, personality traits and sustainability: Construction and empirical application of assessment indicators for attractive attributes of China-chic T-shirt products. Frontiers in Psychology. 2022;13: 1101978. doi: 10.3389/fpsyg.2022.1101978 36643704 PMC9838771

[82] Fu GY, Wang Y, Wang T, Feng SB, Lv H. Selection analysis of BIM forward collaborative design platform based on fuzzy analytic hierarchy process. Railroad Computer Application. 2022;31: 43–49. doi: 10.3969/j.issn.1005-8451.2022.01.07

[83] Kaleli C, Özlü T. The life cycle of a cultural heritage tourism destination: The case of Boğazkale. lnternational Journal of Geography and Geography Education. 2024;51: 68–84. doi: 10.32003/igge.1375082

[84] Xie Y, Hirabayashi S, Hashimoto S, Shibata S, Kang J. Exploring the spatial pattern of urban Forest ecosystem services based on i-tree eco and spatial interpolation: a case study of Kyoto City, Japan. Environmental Management. 2023;72(5): doi: 10.1007/s00267-023-01847-4 37382645

[85] Mastervich B, Garbach K, Harwell MC. Enhancing multiple benefits of brownfield cleanups by applying ecosystem services concepts. Frontiers in Ecology and Evolution. 2024;12: 1286150. doi: 10.3389/fevo.2024.1286150 38487592 PMC10936587

[86] Preston PD, Dunk RM, Smith GR, Cavan G. Examining regulating ecosystem service provision by brownfield and park typologies and their urban distribution. Urban Forestry & Urban Greening. 2024;95: 128311. doi: 10.1016/j.ufug.2024.128311

[87] Russo A. Renaturing for Urban Wellbeing: A Socioecological Perspective on Green Space Quality, Accessibility, and Inclusivity. Sustainability.2024;16(13): doi: 5751.10.3390/su1613575

[88] Becerra M. Gentrifying force or a force for environmental justice? A national assessment of brownfield redevelopment and gentrification in the United States from 2006 to 2015. American Behavioral Scientist. 2024;68(4): 486–502. doi: 10.1177/00027642221140839 PMC12346729 40809922

[89] Ramezani M, Tabrizi AB, Rahmani EK, Campisi T. Identifying the Features of a Walkable-Oriented Redevelopment of Brownfields: A Systematic Review. In International Conference on Innovation in Urban and Regional Planning; 2023 September 6-8; L’Aquila, Italy. Springer, Cham; 2024. p. 447–459. doi: 10.1007/978-3-031-54096-7_39

[90] Pellicelli G, Rossetti S, Zazzi M. A smart and active mobility assessment protocol for urban regeneration. Application to regeneration projects of medium-sized cities in Emilia-Romagna. TeMA-Journal of Land Use, Mobility and Environment. 2024;07: 53–66. doi: 10.6093/1970-9870/10910

[91] Chang SI. Smart city case study through urban regeneration living lab in the information technology era: Focusing on “Tech City” in London, England. 혁신기업연. 2024;9(1): 261–282. doi: 10.37297/IER.2024.3.9.1.261

