# Supplementary material for: Assessment of potential dominant factors for brownfield landscape regeneration: A case study in Xi’an, China
Source: PLoS One. 2025 Feb 10;20(2):e0312668. doi: 10.1371/journal.pone.0312668 (PMC11809921; doi:10.1371/journal.pone.0312668)
Supplement: S1 Appendix — Relevant studies analyzed the map source of the figure and redesigned it based on the original base map. Involves on-site photographs taken during the research and a copyright statement for the design drawings. (DOCX) [file pone.0312668.s001.docx]

**Appendix I. Figure Analyzing Maps Source and copyright statement**

**(1). Figure Analyzing Maps Source （Fig 1）**：

<https://eol.jsc.nasa.gov/SearchPhotos/CoordinateRangeSearch.pl?UpperLat=34.2&daytime=on&HasCloudMask=on&UseCatalogedWithoutCP=on&nighttime=on&UseNotCataloged=on&RightLon=109.1&IncludeHO=on&dawndusk=on&LeftLon=109.1&UseCatalogedWithCP=on&LowerLat=34.2>

**(2). Figure copyright statement:**

**Fig 1：**A figure is similar but not identical to the original image and is therefore for illustrative purposes only.

**Fig 2-Fig 5:** It's a drawing of the result of my own design, No copyright issues.

**Fig 6:** site pictures are self-taken, No copyright issues.

**Fig 7:** It's a drawing of the result of my own design, No copyright issues.
