# Supplementary material for: Assessment of potential dominant factors for brownfield landscape regeneration: A case study in Xi’an, China
Source: PLoS One. 2025 Feb 10;20(2):e0312668. doi: 10.1371/journal.pone.0312668 (PMC11809921; doi:10.1371/journal.pone.0312668)
Supplement: S1 Raw images — (PDF) [file pone.0312668.s002.pdf]

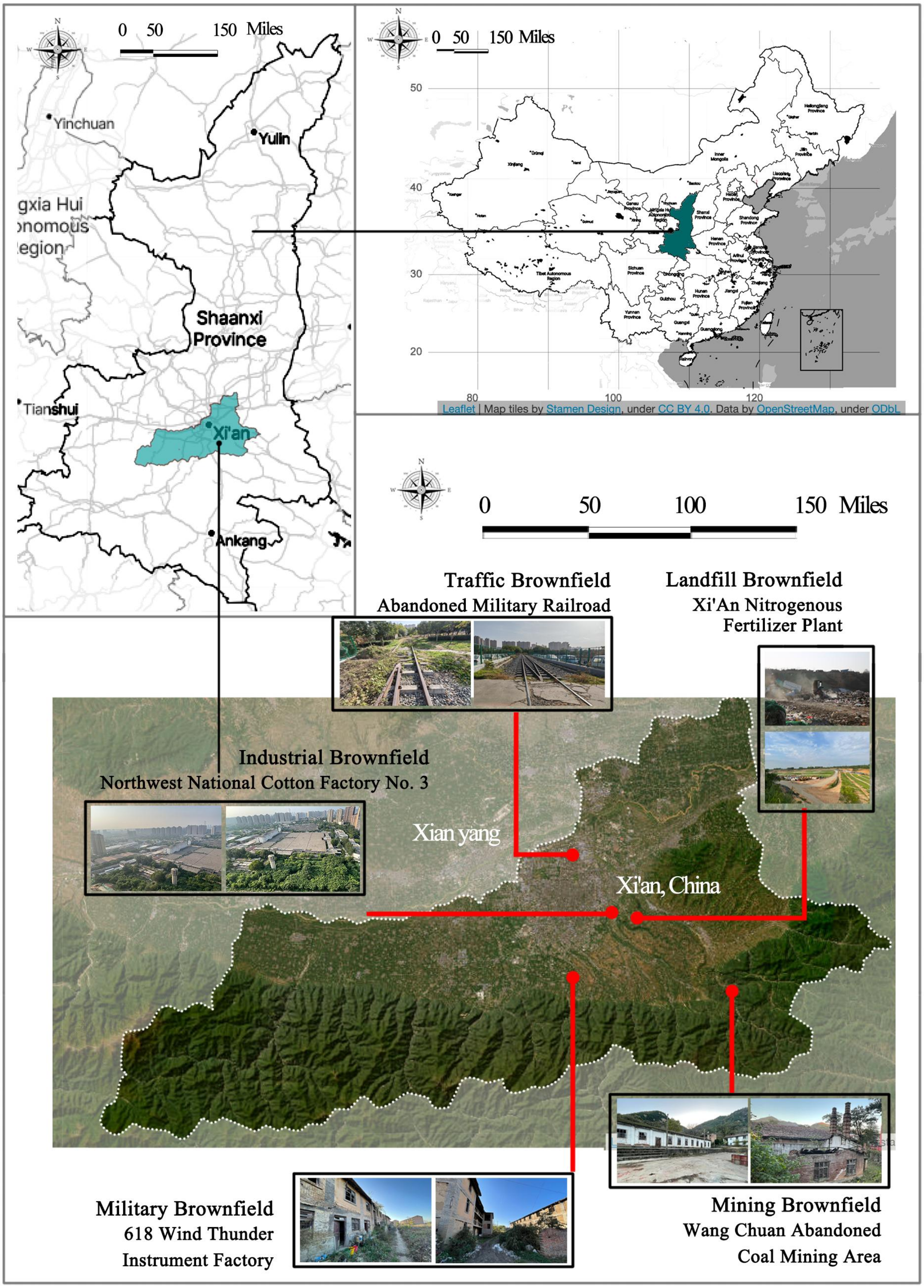

Fig 1. Location of Xi'an, China, main distribution areas of brownfields, and siting of five types of brownfields.

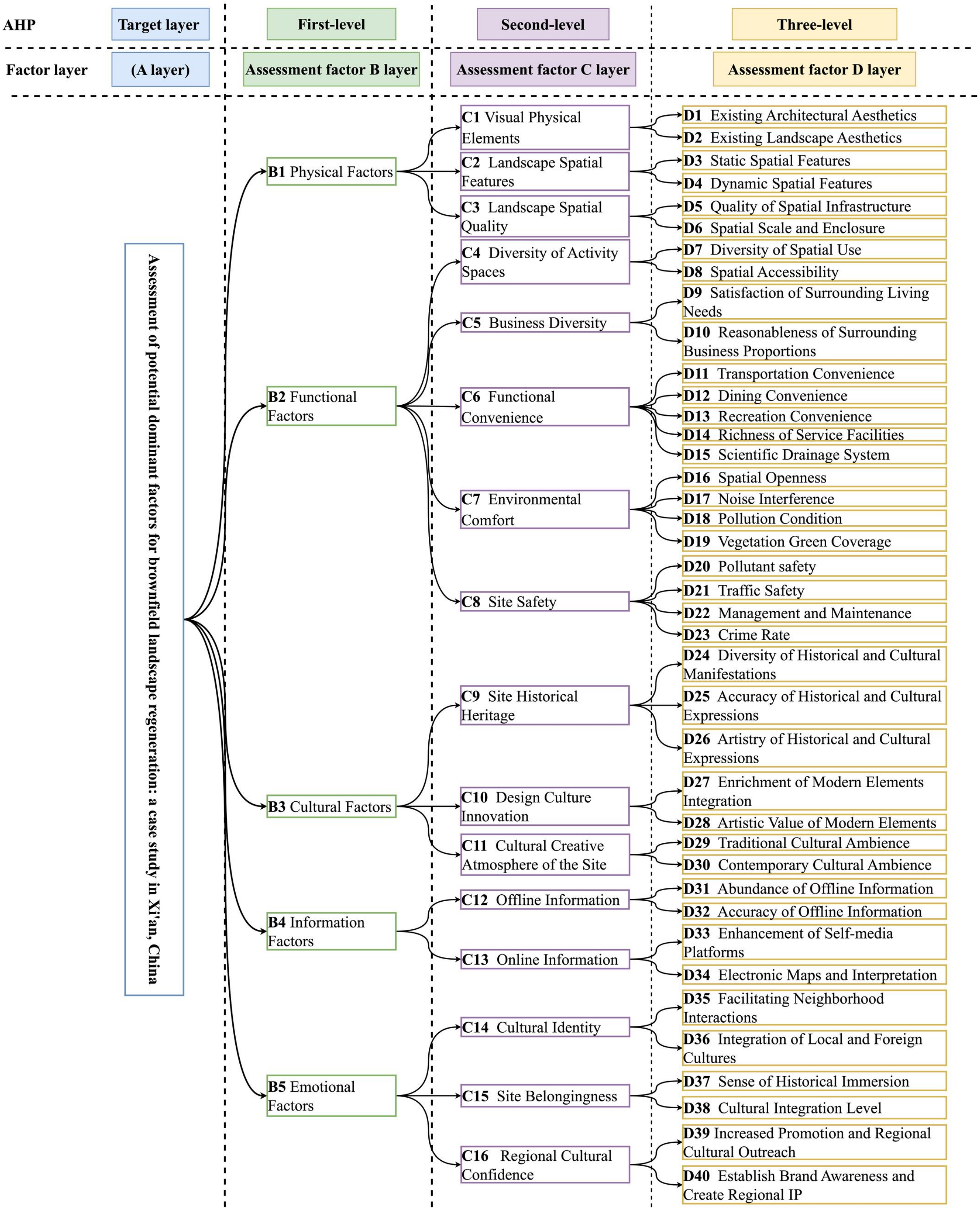

Fig 2. Assessment indicator AHP model for brownfield landscape regeneration in Xi'an.

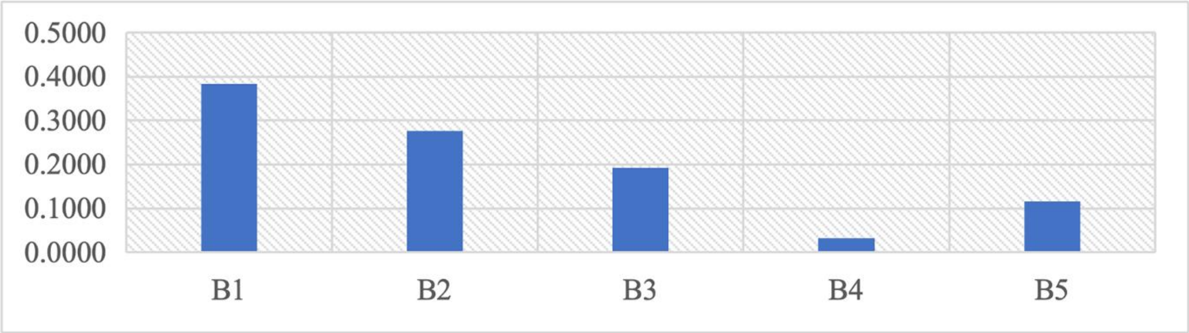

Fig 3. Weights of the first-layer assessment factors in Layer B.

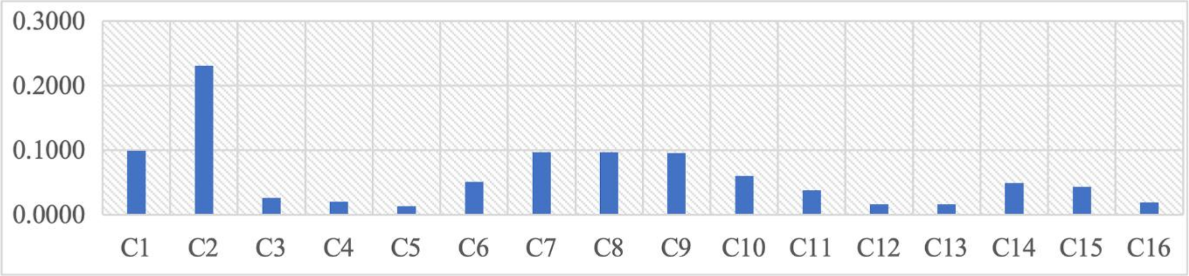

Fig 4. Comprehensive weight of Layer C in second-layer assessment factors.

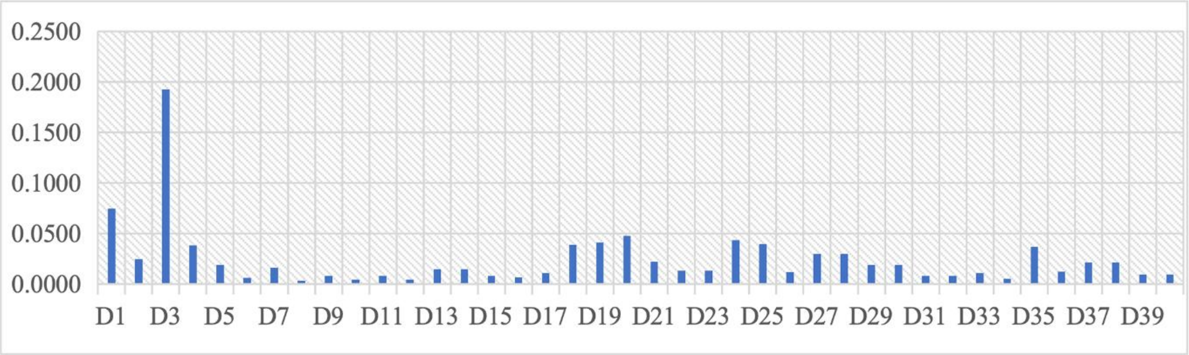

Fig 5. Comprehensive weight of Layer D.

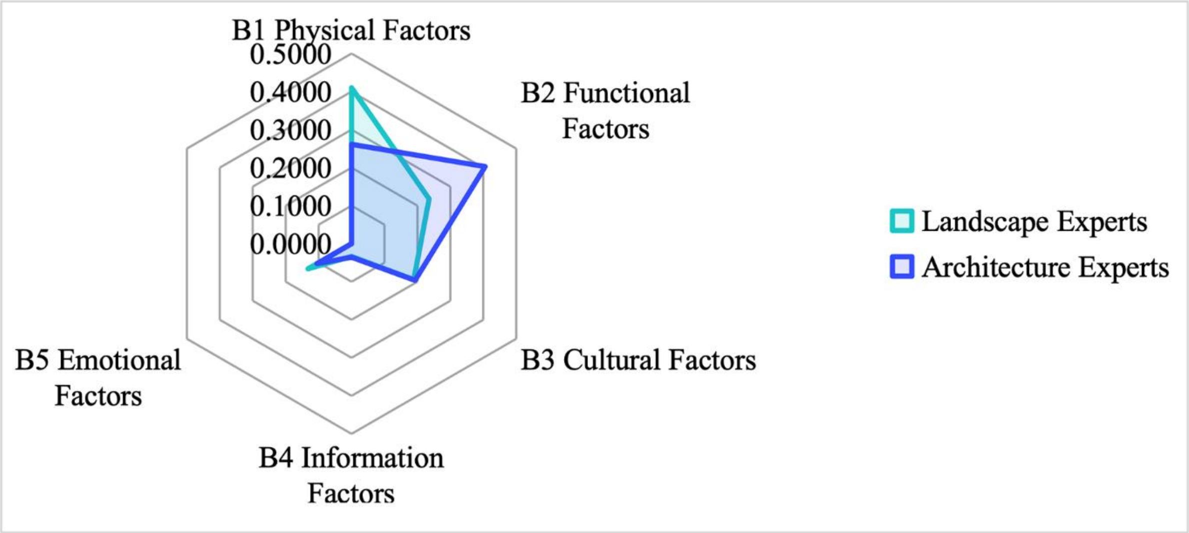

Fig 6. Differences in importance assessment among different experts.

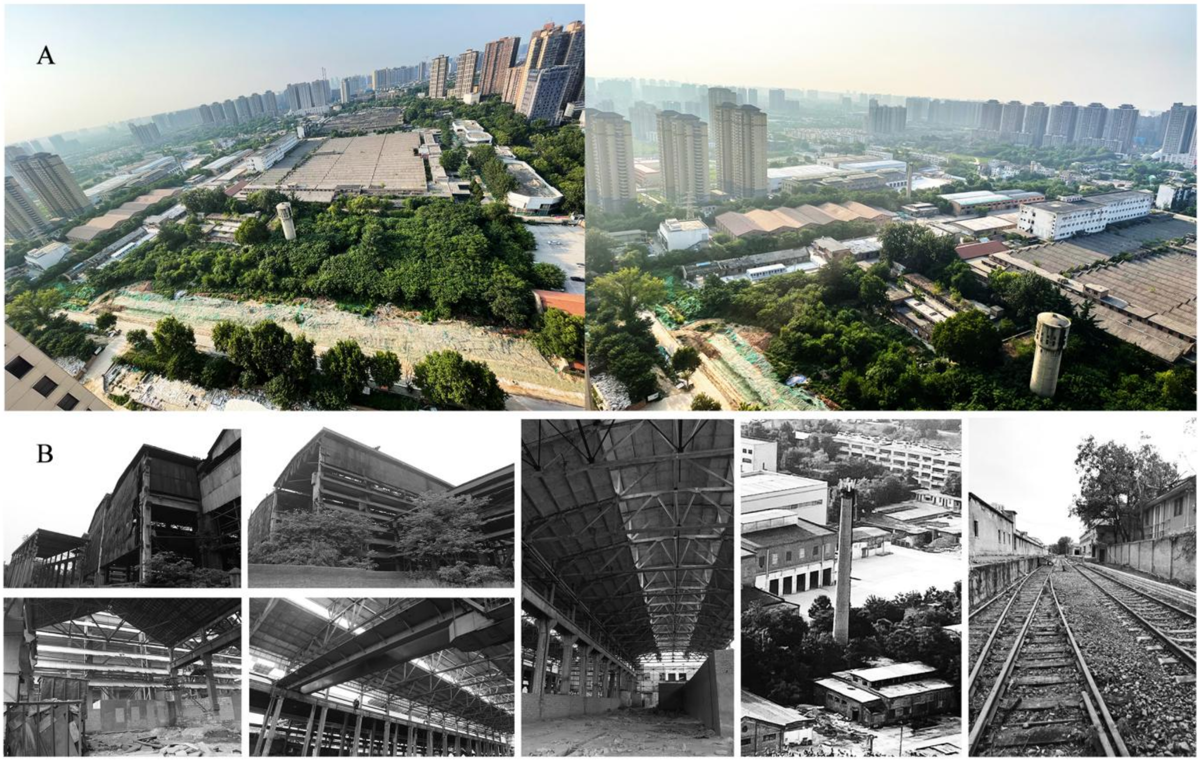

Fig 7. Site spatial-physical status and industrial heritage construction.

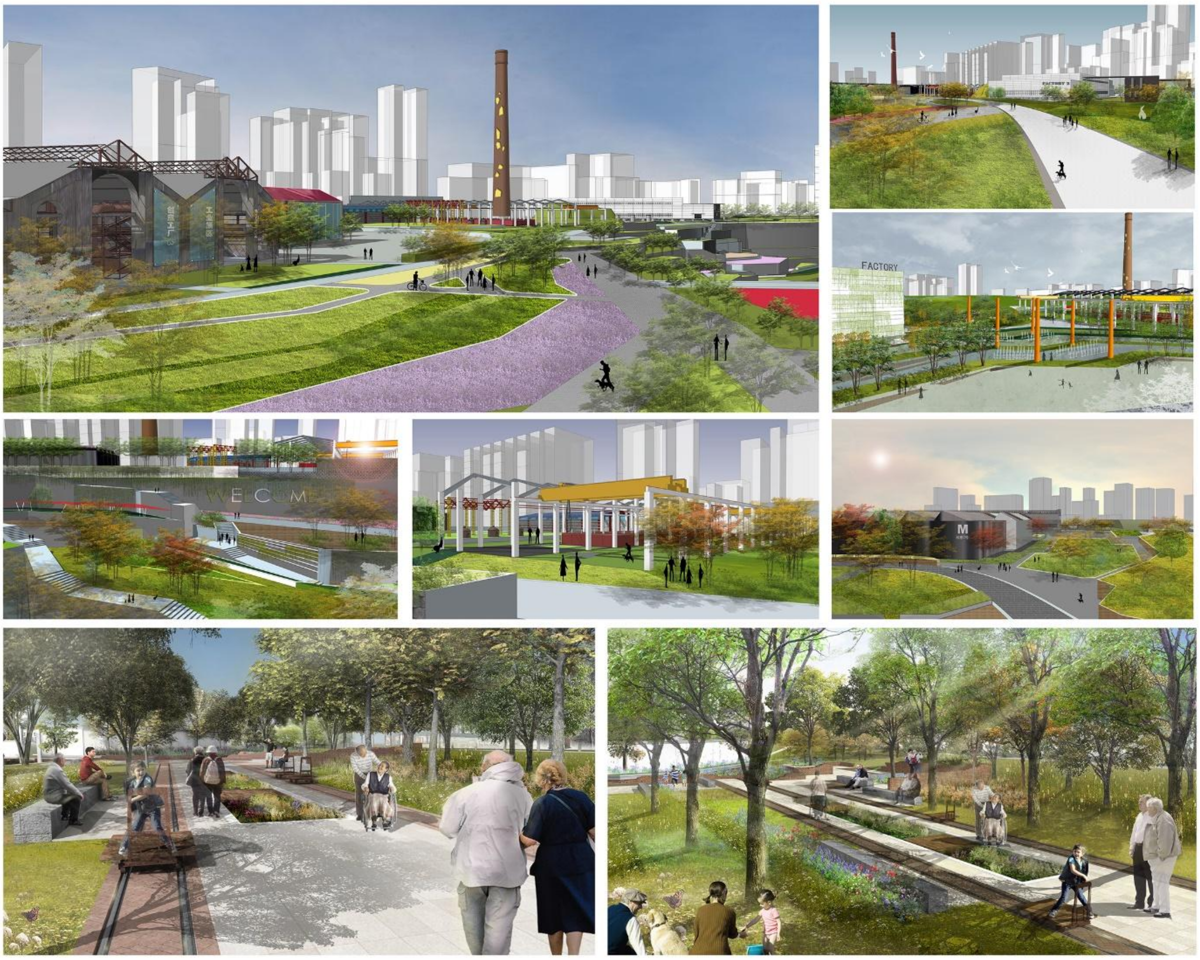

Fig 8. Landscape regeneration of industrial buildings, structures, terrain, and plants.
